# Who Does the Job? How Copper Can Replace Noble Metals in Sustainable Catalysis by the Formation of Copper–Mixed Oxide Interfaces

**DOI:** 10.1021/acscatal.2c01584

**Published:** 2022-06-14

**Authors:** Christoph W. Thurner, Nicolas Bonmassar, Daniel Winkler, Leander Haug, Kevin Ploner, Parastoo Delir Kheyrollahi Nezhad, Xaver Drexler, Asghar Mohammadi, Peter A. van Aken, Julia Kunze-Liebhäuser, Aligholi Niaei, Johannes Bernardi, Bernhard Klötzer, Simon Penner

**Affiliations:** † Department of Physical Chemistry, University of Innsbruck, Innrain 52c, A-6020 Innsbruck, Austria; ‡ Max Plank Institute for Solid State Research, Heisenbergstaße 1, D-70569 Stuttgart, Germany; § Reactor & Catalyst Research Laboratory, Department of Chemical and Petroleum Engineering, University of Tabriz, 29 Bahman Boulevard, Tabriz 51666-16471, Iran; ∥ University Service Centre for Transmission Electron Microscopy (USTEM), Technische Universität Wien, Wiedner Hauptstraße 8-10/057-02, A-1040 Wien, Austria

**Keywords:** copper, exsolution, interface, metal−mixed oxide, NO abatement, N_2_O, palladium, perovskite

## Abstract

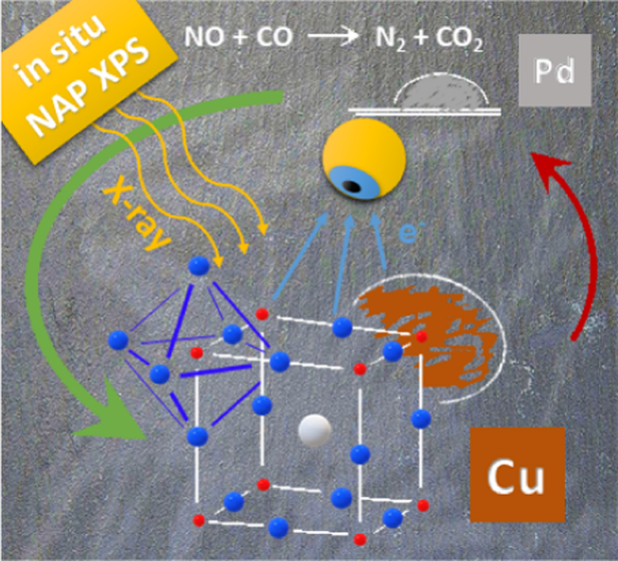

Following the need for an innovative catalyst and material design in catalysis, we provide a comparative approach using pure and Pd-doped LaCu*_x_*Mn_1–*x*_O_3_ (*x* = 0.3 and 0.5) perovskite catalysts to elucidate the beneficial role of the Cu/perovskite and the promoting effect of Cu*_y_*Pd*_x_*/perovskite interfaces developing *in situ* under model NO + CO reaction conditions. The observed bifunctional synergism in terms of activity and N_2_ selectivity is essentially attributed to an oxygen-deficient perovskite interface, which provides efficient NO activation sites in contact with *in situ* exsolved surface-bound monometallic Cu and bimetallic CuPd nanoparticles. The latter promotes the decomposition of the intermediate N_2_O at low temperatures, enhancing the selectivity toward N_2_. We show that the intelligent Cu/perovskite interfacial design is the prerequisite to effectively replace noble metals by catalytically equally potent metal–mixed-oxide interfaces. We have provided the proof of principle for the NO + CO test reaction but anticipate the extension to a universal concept applicable to similar materials and reactions.

## 1 Introduction

Innovative knowledge-based catalyst design as a prerequisite to account for the complexity of catalytic reactions increasingly centers at the intersection of both heterogeneous catalysis and materials chemistry. Simple catalyst preparation is more and more replaced by following sophisticated synthesis pathways guided by and exploiting the physicochemical and structural properties of materials. This concept is valid both for various material classes (e.g., intermetallic compounds or complex oxides) and for a variety of catalytic reactions, such as methanol steam and methane dry reforming, CO oxidation, de-NO*_x_* catalysis, water–gas-shift reaction, or CO_2_ hydrogenation.^1−15^ A very promising pathway is the *in situ* decomposition of precursor materials in the respective reaction mixture and the formation of metal–oxide interfaces accounting for the bifunctional mechanism prevalent in more complex reactions.^4,7^ The formation of such interfaces is a recurrent theme for many materials that are seemingly structurally and chemically different in their precursor states. Depending on the degree of decomposition and the chemical nature of the constituting catalyst building blocks, either simple metal–oxide or structurally more complex metal–(partially) decomposed precursor interfaces result. To exemplify the concept, it has been recently shown that the decomposition of Cu–Zr and Cu–In intermetallic compounds in methanol steam or Pd–Zr methane dry reforming mixtures caused the *in situ* formation of Cu–ZrO_2_, Pd–ZrO_2_, or Cu*_x_*In*_y_*/In_2_O_3_ interfaces with different materials and catalytic properties.^6,9,13,15^ Similarly, complex ternary oxides such as perovskites (ABO_3_) feature the formation of metal–oxide or metal–mixed-oxide interfaces. This is especially valid for doped perovskite materials and has been worked out in detail on La–Ni perovskites in methane dry reforming.^2,4,11^ Furthermore, it has been recently demonstrated in the catalytic reduction of NO by CO that the metal–oxide (i.e., Pd-perovskite) interfaces result from the decomposition of Pd-doped lanthanum iron manganites significantly differing in their properties from interfaces that were deliberately prepared by, e.g., conventional impregnation.^7^ For such perovskite materials, the decomposition concept is very much tied to the metal exsolution from the perovskite lattice. Controlling this exsolution by exploiting the properties of the entire perovskite entity and the individual building blocks allows for direct steering of the interfacial properties of the resulting metal–oxide or metal–mixed-oxide material. The so-formed interfaces derived from perovskites usually combine two beneficial mechanistic aspects: the oxygen-vacancy chemistry of the oxide promoting dissociation of O-containing gas-phase molecules^16−18^ and the metal–d-band structure fulfilling proper adsorption chemistry for the activation or release of selected gas-phase species.^10,19,20^ Thereby, both the oxide and metallic constituents can be tuned by dopants to change the oxygen-vacancy reactivity^5,21,22^ or to alter the d-band structure^23,24^ and thus the adsorption properties.

Initially, the exsolution concept was demonstrated in 2002 by Nishihata et al. who studied a LaFe_0.57_Co_0.38_Pd_0.05_O_3−δ_ perovskite catalyst in the reduction of NO by CO.^25^ Their results showed that exsolution of Pd and Co nanoparticles enhanced the long-term stability, in contrast to significant activity loss over time on a Pd-impregnated γ-Al_2_O_3_ catalyst. Due to the anchored nature of such exsolution catalysts, the surface diffusion is effectively suppressed, hindering particle agglomeration.^26,27^

In the upcoming years, exsolution of various monometallic and bimetallic nanoparticle catalysts from different perovskites have already been successfully demonstrated in combining the benefits of metal–mixed-oxide interfaces and in improving the efficiency of gas conversion processes involving redox reactions. In this context, the concept has been successfully transferred to several perovskites and precious metals; Pt and Rh exsolution from CaTiO_3−δ_^28−30^ and Pd exsolution from LaFeO_3−δ_,^31−33^ BaCeO_3−δ_,^34−36^ or YFeO_3−δ_^37^ is thereby common, under a variety of applied atmospheres. The concept of reversible exsolution/reintegration into the perovskite lattice, crucial for catalyst self-regeneration during catalytic cycling, has also been highlighted.^27^ Therefore, perovskites are considered as a more economical and more abundant alternative to supported metal catalysts.^38,39^ In this perspective, the Pd/perovskite interface was suggested as a particularly promising candidate in promoting the NO + CO reaction.^7,25,33^

As shown by various studies, the reduction of NO by CO (1) is a versatile test reaction to investigate the thermocatalytic behavior of metal–mixed-oxide interfaces resulting from metal exsolution.^25,27,32,33^ Metal–mixed-oxide interfaces provide distinct adsorption sites for the dissociative activation of NO, aided by vacancies in oxygen-deficient metal–oxides^38,40,41^ and perovskites.^39^ In contrast, CO adsorption is favorable on metallic sites and helps in regenerating surface oxygen vacancies by adsorption from the gas phase and subsequent reaction at the interface.^39^ The resulting sum reaction is
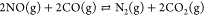
1Metal exsolution from dedicated perovskites also allows steering the formation/decomposition of N_2_O as a transient species resulting from partial reduction of NO (2)^42,43^
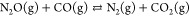
2In summary, within the framework of the NO + CO reaction, the exsolution concept allows us to create highly reactive and anchored active metal/metal–oxide sites under *in situ* reaction conditions. In this perspective, LaMnO_3_ as a stable and active parent structure^44,45^ is considered as the perfect model perovskite structure to highlight the advantages of controlled decomposition and is therefore chosen as the perovskite starting material. In addition, we take advantage of the substitution of manganese by copper and palladium in LaMnO_3_, generating oxygen defects,^46,47^ which are supposed to serve as binding sites for NO activation. Recent theoretical calculations indicate that Cu doping increases the intrinsic reducibility of LaMnO_3_.^48^^48^ The same argumentation holds for Pd as the reducibility of palladium oxide exceeds that of copper oxide.^49^ In addition, Pd is prone to alter the d-band structure of Cu by alloying. Consequently, in promoting the reductive exsolution of (bi-)metallic particles (e.g., Cu and Cu*_y_*Pd*_x_*), the addition of Cu/Pd is expected to induce the formation of a potentially active vacancy-doped Cu/perovskite or Cu*_y_*Pd*_x_*/perovskite interface. Regarding the decomposition of N_2_O, metallic Cu is also considered as a highly active catalyst in facilitating this reaction pathway, shifting the selectivity toward N_2._^50,51^

Within this work, we show how the controlled exsolution of Cu (and Pd) from pure and Pd-doped LaCu*_x_*Mn_1–*x*_O_3−δ_ perovskite structures leads to the formation of synergistically acting Cu/perovskite interfaces that in essence allow replacing the noble metal Pd by copper without compromising the activity for NO reduction by CO. Following this approach, both the promotional effects of Pd and the catalytic effect of the Cu content can be investigated, directly followed by the synthesis of pure LaCu*_x_*Mn_1–*x*_O_3−δ_ (*x* = 0.3 and 0.5; termed LCM37 and LCM55 in the following) perovskite catalysts and their respective B-site-doped variants La(Cu*_x_*Mn_1–*x*_)_1–y_Pd_y_O_3−δ_ (*y* = 0.004 and 0.02) with 0.18 and 0.86 wt % Pd. The corresponding establishment of structure–activity/selectivity relationships is particularly aided by *in situ* near-ambient pressure X-ray photoelectron spectroscopy (NAP-XPS) experiments in the NO + CO reaction mixture, which allows us to exactly pinpoint the onset of Cu exsolution and to correlate the reactivity of the transient N_2_O species with the appearance of Cu at the surface following exsolution during operation. Especially, the presented LCM55 may represent a promising catalyst lead material to economize the use of noble metals.

## 2 Experimental Section

### 2.1 Synthesis of the Materials

The perovskite catalysts La(Cu*_x_*Mn_1–*x*_)_1–*y*_Pd*_y_*O_3−δ_ (*x* = 0.3 and 0.5; *y* = 0, 0.004, and 0.02) were prepared by a sol–gel self-combustion method. The stoichiometric amounts of metal nitrates were dissolved in deionized water. As a complexing agent, glycine was added (molar ratio of glycine/nitrate = 1). The solution was then heated to 80 °C under continuous stirring to evaporate the excess of water until a sticky gel was obtained. To carry out gel decomposition, the temperature was raised to 250 °C, and finally, the gel self-ignited, yielding a black powder (total yield of about 90%). As a final step, the powder was calcined at 700 °C for 5 h in the air in a muffle furnace. As starting materials, La(NO_3_)_3_·6H_2_O (Alfa Aesar, 99.9% (REO), 433.01 g mol^–1^), Mn(NO_3_)_2_·4H_2_O (Sigma-Aldrich, 97.0% (KT), 251,01 g mol^–1^), Cu(NO_3_)_2_·3H_2_O (Merck, 99.5%, 241,60 g mol^–1^), Pd(NO_3_)_2_·2H_2_O (Sigma-Aldrich, 99.9%, 266.46 g mol^–1^), and glycine (Sigma-Aldrich, 99.9%, 75.07 g mol^–1^) were used.

As the verification of the synergistically operating Cu/perovskite interface requires proper referencing to Cu-free, Pd-free, and perovskite-free materials, we have used the respective catalysts either in a commercially available form or synthesized them accordingly. For the 2.0 wt % Pd on an alumina reference catalyst, a commercial material from Heraeus Hanau was used. The 7.0 wt % Cu on the silica reference catalyst was synthesized by a wet impregnation approach. SiO_2_ powder (Alfa Aesar, 99.0% (metals basis), 60.08 g mol^–1^) was suspended in an aqueous solution (6 g L^–1^) of copper acetate (Merck, 99%, 181.63 g mol^–1^), with the solvent being subsequently slowly removed in a rotary evaporator. The bluish solid was then calcined at 400 °C for 2 h in the air to yield the gray-colored catalyst with a loading of 7.0 wt % of metallic copper after prereduction (350 °C, 3 h, 5% H_2_ in He).

### 2.2 Structural and Spectroscopic Characterization

#### 2.2.1 Transmission Electron Microscopy (TEM)

High-resolution transmission electron microscopy (HRTEM) and energy-dispersive X-ray spectroscopy (EDXS) were carried out at 200 kV with a JEOL JEM-ARM200F TEM equipped with a cold field-emission gun, a Gatan Imaging Filter Quantum ER, and a C_s_ corrector (CETCOR, CEOS GmbH). HRTEM images were collected via a Gatan Ultrascan XP1000 camera, and EDXS elemental mappings were performed with a JEOL JED-2300 DrySDTM detector. For the measurements, the catalysts were suspended in isopropanol and transferred onto a gold grid via drop casting.

#### 2.2.2 Powder X-ray Diffraction (PXRD)

*Ex situ* XRD measurements were performed in the transmission mode, utilizing a Stadi P diffractometer (STOE & Cie GmbH, Darmstadt, Germany). This setup is equipped with a MYTHEN2 DCS4 detector (DECTRIS Ltd., Switzerland) and a Mo X-ray tube (GE Sensing & Inspection Technologies GmbH, Ahrensburg, Germany). A curved Ge(111) crystal selects the Mo Kα_1_ radiation with a wavelength of 0.7093 Å. The analysis of the diffractograms was carried out with the software WinXPOW using reference data from the ICDD and ICSD databases. References from ICDD are given below including the chemical formula and the card number: Cu, 00-004-0836;^52^ La_2_O_3_, 00-005-0602;^53^ and La_2_CuO_4_, 00-030-0487;^54^ References from ICSD are given below including the chemical formula and the database code: LaCu_0.5_Mn_0.5_O_3_, 193762;^55^ LaCu_0.3_Mn_0.7_O_3_, 92191.^47^

#### 2.2.3 Specific Surface Area (BET)

BET measurements were performed with a NOVA 2000e Surface Area & Pore Size Analyzer (Quantachrome Instruments) using the software Quantachrome NovaWin. Sample pretreatment involved heating to 200 °C *in vacuo* for 30 min followed by adsorption of N_2_ at −196 °C at five points from 0.05 to 0.30 p/p_0_.

#### 2.2.4 *Ex Situ* X-ray Photoelectron Spectroscopy (XPS)

The chemical state of the sample surface was investigated with XPS utilizing a Thermo Scientific MultiLab 2000 spectrometer. It is equipped with a monochromatic Al Kα X-ray source and an α 110 hemispherical sector analyzer. All recorded core-level regions were referenced to the “adventitious” C 1s peak at 284.8 eV. Generally, the spectra were recorded after transfer in ambient air under UHV conditions (10^–9^ mbar) at a pass energy of 20 eV.

#### 2.2.5 Near-Ambient Pressure X-ray Photoelectron Spectroscopy (NAP-XPS)

To elucidate the sample’s surface state under *in situ* reaction conditions, experiments in a commercial UHV system for NAP-XPS applications (SPECS GmbH) were carried out. The UHV chamber is comprised of a μFOCUS 600 NAP monochromatic small spot (100 × 300 μm^2^) Al Kα X-ray source, a hemispherical energy analyzer (PHOBIOS 150 NAP) in a vertical configuration, and a μ-metal analyzing chamber, which shields the system from external magnetic fields. The differentially pumped energy analyzer allows backfilling of the analyzing chamber to pressures up to 30 mbar with different gases and gas mixtures (e.g., NO + CO) via mass flow controllers (Bronkhorst). To investigate the powdered samples, a pressed pellet covering a stainless steel grid as a stabilizer is fixed on a sample holder by mounting the pellet via a front plate. An IR laser (IPG PHOTONICS, 100 W max. power) is attached to the bottom side of the analyzing chamber and allows us to heat the samples from the back side via an 8 mm hole in the sample holder. The temperature is controlled by a K-type thermocouple fixed on the stainless steel grid inside the pellet. During all of the experiments, the atmosphere consisting of a NO and CO (ratio 1:1; 0.3 mbar each; CO: Messer, purity 4.7; NO: Linde, purity 2.5) gas mixture was utilized. The exited photoelectrons were collected by a 300 μm nozzle directly from the sample’s frontside surface via an 8 mm opening in the front plate. Due to pressures in the mbar regime, the X-ray ionized gas region between the nozzle and the sample compensates charging, thus a core-level shift can be neglected even on poorly conducting samples. The X-ray source power was set to 70 W and 13 kV, and all spectra were recorded under the exactly same conditions, especially regarding the pass energy settings (50 eV).

#### 2.2.6 Semiquantitative Evaluation of Reaction Kinetics and Particle Coverage from XPS

To determine the activation energy (E_a_) and preexponential factor, the catalytic data (i.e., initial consumption rate at low temperatures) were fitted via an Arrhenius fit function *A*·exp(−*E*_a_/(*R*·*T*)). In addition, the particle coverage was estimated by analyzing the XPS data (La 3d and Cu 2p) via an attenuated overlayer model^56^ extended for fractional coverage by particles. For more details, see Supporting Information Appendix A, Figure S1 and Tables S1 and S2.

### 2.3 Catalytic Experiments in NO Reduction by CO

We investigated the catalytic properties of pure LaCu_0.3_Mn_0.7_O_3−δ_ (LCM37), pure LaCu_0.5_Mn_0.5_O_3−δ_ (LCM55), and their respective B-site-doped variants with 0.18 and 0.86 wt % Pd. For this, the catalysts (200 mg) were fixed with quartz wool (bed length: 25 mm) in a quartz tube (i.d. 8 mm) fixed-bed flow reactor setup. The catalysts were heated at 2 °C min^–1^ from 50 °C up to 500 °C in a total volumetric gas flow of 200 mL min^–1^ (GHSV = 9.6 × 10^3^ h^–1^) in a gas mixture of 1% NO and 1% CO and using He as the carrier gas. To avoid parasitic reactions from a thermocouple inside the reactor, an independent calibration of the exact gas temperature in the equivalent flow of pure helium was performed by placing a K-type thermocouple exactly at the location of the catalyst bed. To track the number of educts, products, and intermediates continuously, the downstream gas was analyzed online both by a quadrupole mass spectrometer (Balzers QMA 125) and an FT-IR spectrometer (Agilent Cary 600 series) equipped with a gas cell. Details of the catalytic data evaluation are given in Supporting Information Appendix B, Figure S2 and Tables S3 and S4.

## 3 Results and Discussion

### 3.1 Structural and Spectroscopic Consequences of Cu and Pd Exsolutions from the LaCu*_x_*Mn_1–*x*_O_3_ Lattice

The catalyst’s bulk and surface structure, as well as the chemical composition, were monitored as a function of Pd doping and catalytic treatment for pure and 0.86 wt % Pd-doped LCM37 and LCM55. The catalysts with 0.18 wt % Pd were prepared to show the effect of a low Pd content in the catalytic performance. Due to detection limits resulting from the low amount of Pd (0.18 wt %), an extended catalyst characterization was not carried out.

**Figure 1:**
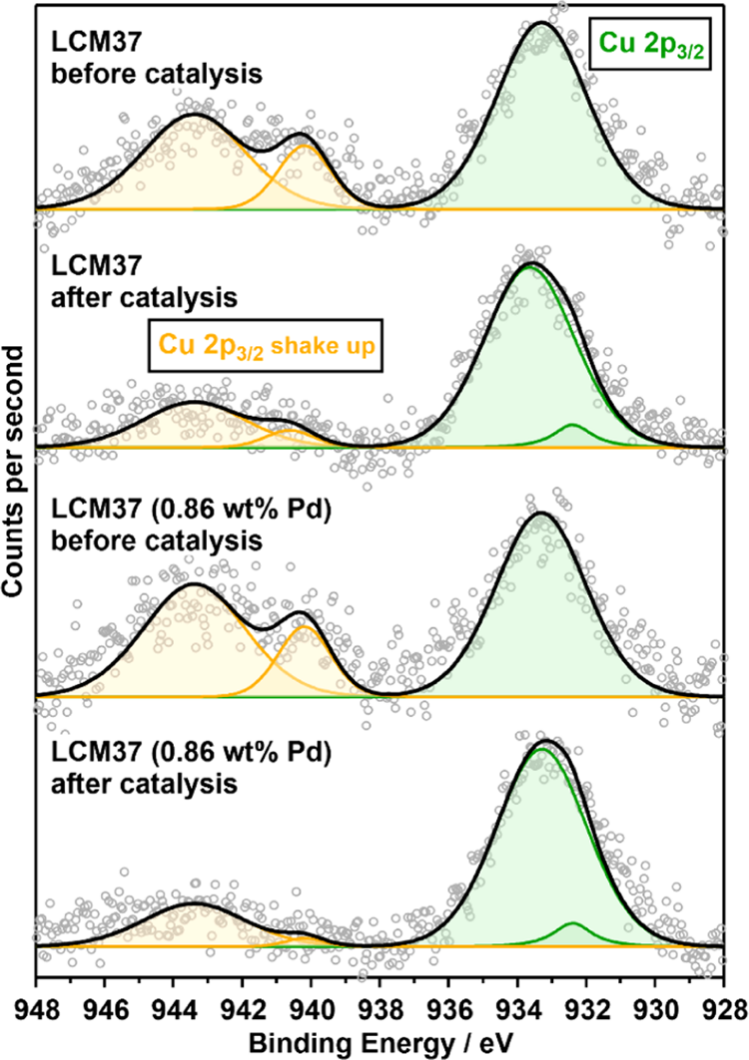
Characterization of the surface compositions of LCM37 and Pd-LCM37 before and after the NO + CO reaction by *ex situ* XPS. The Cu 2p_3/2_ region (gray circles) was deconvoluted into individual metallic and oxidized Cu components (orange trace: Cu(II) in the shake-up feature; green trace: Cu(II) and Cu(0)/Cu(I) in the main peak; fit envelope: black).

Apart from state-of-the-art microscopic investigations, the successful synthesis and incorporation of Pd into the LaCu*_x_*Mn_1–*x*_O_3_ lattice for the LCM37 catalyst was verified by *ex situ* PXRD experiments. The diffractograms in the Supporting Information Appendix C, Figure S3 only reveal peaks of the parent perovskite structure and also for the Pd-doped materials. The exsolution of Cu(0) under reaction conditions at the surface was subsequently directly verified on the LCM37-based catalysts by monitoring the Cu 2p region before and after catalysis using *ex situ* X-ray photoelectron spectroscopy (XPS) (Figure 1). A peak-fitting procedure (Supporting Information Appendix D, Figure S4 and Table S5) of the Cu 2p_3/2_ region^57,58^ allows us to calculate Cu(II) to Cu(0) + Cu(I) proportions on the catalyst surface by comparing the area of the shake-up feature (orange traces at ≈942 eV), representing only Cu(II), and the main Cu 2p_3/2_ peak (green traces at ≈933 eV) representing Cu(0), Cu(I), and Cu(II). The ratios (1.28 and 1.09) between the shake-up peak area (orange traces) and the area of the main Cu 2p_3/2_ peak (green traces) were determined for pure and Pd-doped LCM37 before catalysis (termed “b.c.”) (Figure 1, first and third spectra from top) and indicate the exclusive surface presence of Cu(II). The comparison with the ratio of the peak areas after catalysis (termed “a.c.”) enables the estimation of the amount of Cu(0)/Cu(I) vs Cu(II) (Supporting Information Appendix D, Table S6). The vanishing of the shake-up feature (orange traces) after the NO + CO treatment in both LCM-based samples (Figure 1, second and fourth spectra from top) indicates a clear increase of the Cu(0)/Cu(I) contribution on the respective catalyst surface due to Cu exsolution.

The bulk structure, however, remained essentially unaltered. *Ex situ* powder X-ray diffraction patterns (PXRD) recorded before and directly after the NO + CO reaction both show a structure- and phase-pure catalyst (Supporting Information Appendix C, Figure S3).

**Figure 2:**
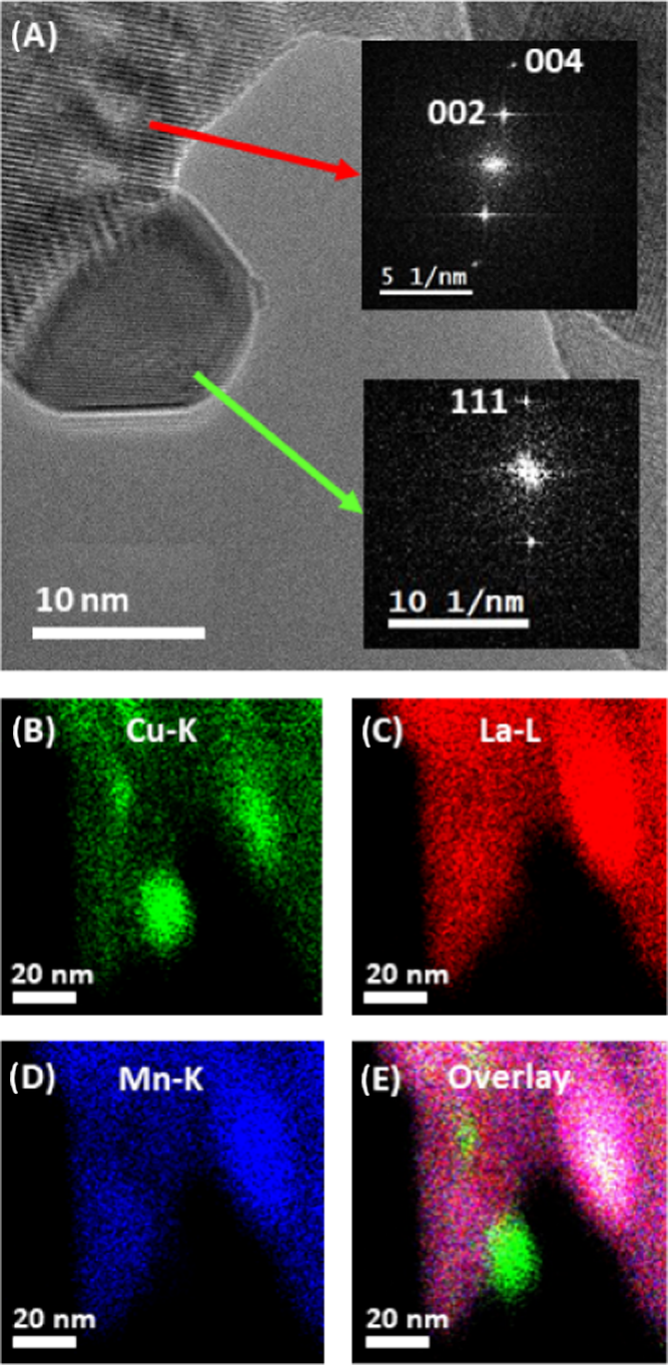
HRTEM and EDXS identical-location experiment of pure LCM37 after NO reduction by CO. (A) An HRTEM image of LCM37 (Fast Fourier Transform shown as upper inset) and that of an isolated exsolved Cu(0) particle (Fast Fourier Transform shown in the lower inset). (B–E) Identical-location EDXS maps highlighting the Cu-K, La-L, and Mn-K intensities. An overlay of the intensities is highlighted in panel (E).

The exsolution of metallic Cu and the formation of small copper nanoparticles from the perovskite structure after applying the reaction mixture were further evidenced by HRTEM and EDXS analysis (Figure 2 and Supporting Information Appendix E, Figures S5–S8). In panel A, a fast Fourier transformation was used to create a locally resolved diffraction pattern (inset) and to distinguish between LCM37 and metallic Cu (Miller indices in brackets). The exsolution of Cu(0) nanoparticles with a size of 5–10 nm was further confirmed by an identical-location EDXS mapping experiment shown in panels B–D, using the Cu-K, La-L, and Mn-K intensities (Panel E shows the overlay of all elements). The large *“crater-shaped”* interface between the exsolved Cu nanoparticle and the perovskite likely stabilizes the Cu particles and prevents them from sintering and oxidative deactivation.^59,60^

**Figure 3:**
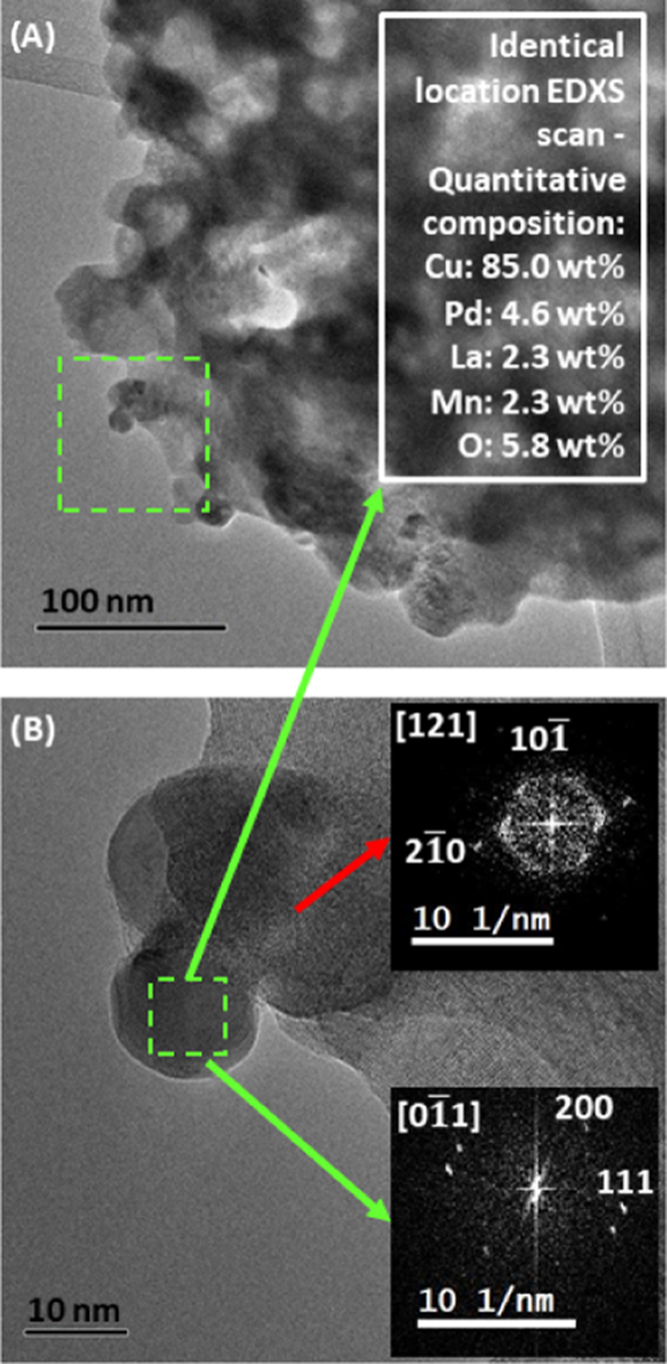
Electron microscopy evaluation of Pd-doped LCM37 after NO reduction by CO. (A) Representative overview bright-field image of Pd-doped LCM37 under bright-field conditions. The area marked with a green square is shown as an HRTEM image in panel (B). (B) Single alloyed copper-rich Cu–Pd particle attached to an LCM37 grain edge evidenced by Fast Fourier Transformations of the particle and the neighboring LCM37 grain. The white box depicts the result of the identical-location EDXS mapping of the green dashed area in panel (B), highlighting the quantitative compositions of Cu, Pd, La, Mn, and O.

Since the reducibility of palladium oxide is higher than that of copper oxide,^49^ we anticipated preceding, or at least simultaneous, Cu(0)–Pd(0) exsolution for Pd-doped LCM37. We found isolated alloyed copper-rich Cu–Pd nanoparticles on the Pd-doped LCM37 sample after catalysis in the electron microscopy experiments (Figure 3), giving rise to the anticipated Cu(Pd)/perovskite interface after the NO + CO treatment.

To further stretch the Cu incorporation capability of the perovskite and to eventually induce the exsolution of a higher amount of Cu nanoparticles and/or other Cu-rich phases, we deliberately prepared LCM55 catalysts with a higher nominal Cu content. In fact, already after synthesis, metallic Cu reflexes in the PXRD pattern were detected. A further increase of Cu(0) reflexes after the NO + CO treatment was also observed (Supporting Information Appendix F, Figure S9), which indicates facilitated Cu nanoparticle exsolution. An additional reflex found at 2θ = 14.1° can be assigned to a minor amount of the parasitic La_2_CuO_4_ cuprate phase. A small quantity of La_2_O_3_ emerges as a byproduct during Cu exsolution.

Further characterization of the LCM55-based catalysts before catalysis was carried out by HRTEM and EDXS mapping (Supporting Information Appendix F, Figures S10–S13). In addition to homogeneously distributed Cu and Pd in the perovskite lattice, we already observed both pure Cu and Cu/Pd alloy nanoparticles after preparation. XPS measurements of the Cu 2p_3/2_ region confirm the occurrence of metallic Cu particles on the LCM55 surface before catalysis and their further growth during catalysis (Supporting Information Appendix G, Figure S14 and Table S7).

### 3.2 Catalytic Consequences of the Metal Exsolution

Based on our experiences in investigating highly complex systems with dynamic (mixed) metal/mixed-oxide interfaces, we deliberately refrained from the tentative assignment of static, special active sites and, instead, focused on keeping the process parameters such as catalyst quantity and volumetric flow rate as constant and reproducible as possible. Therefore, the determination of the mass-specific activity (mol s^–1^ g^–1^) based on the contact time to the catalyst bed was chosen as the most promising basis for rate/activity calculations. As our samples exhibited no major variation of the BET surface area (see Supporting Information Appendix H, Table S8), normalization to the catalyst mass appeared best arguable over the dubious strategy of assumption-loaded “active site counting”. This argument holds especially true in view of the structural dynamics, progressive alterations, and overall chemical complexity of the distinct catalyst’s surfaces. Kinetic studies and particle coverage estimation were carried out to semiquantitatively compare the reaction kinetics with the active sites (Supporting Information Appendix A, Figure S1 and Tables S1 and S2). For gas-phase analysis and data-evaluation procedure within the catalytic tests, see Supporting Information Appendix B, Figure S2.

**Figure 4:**
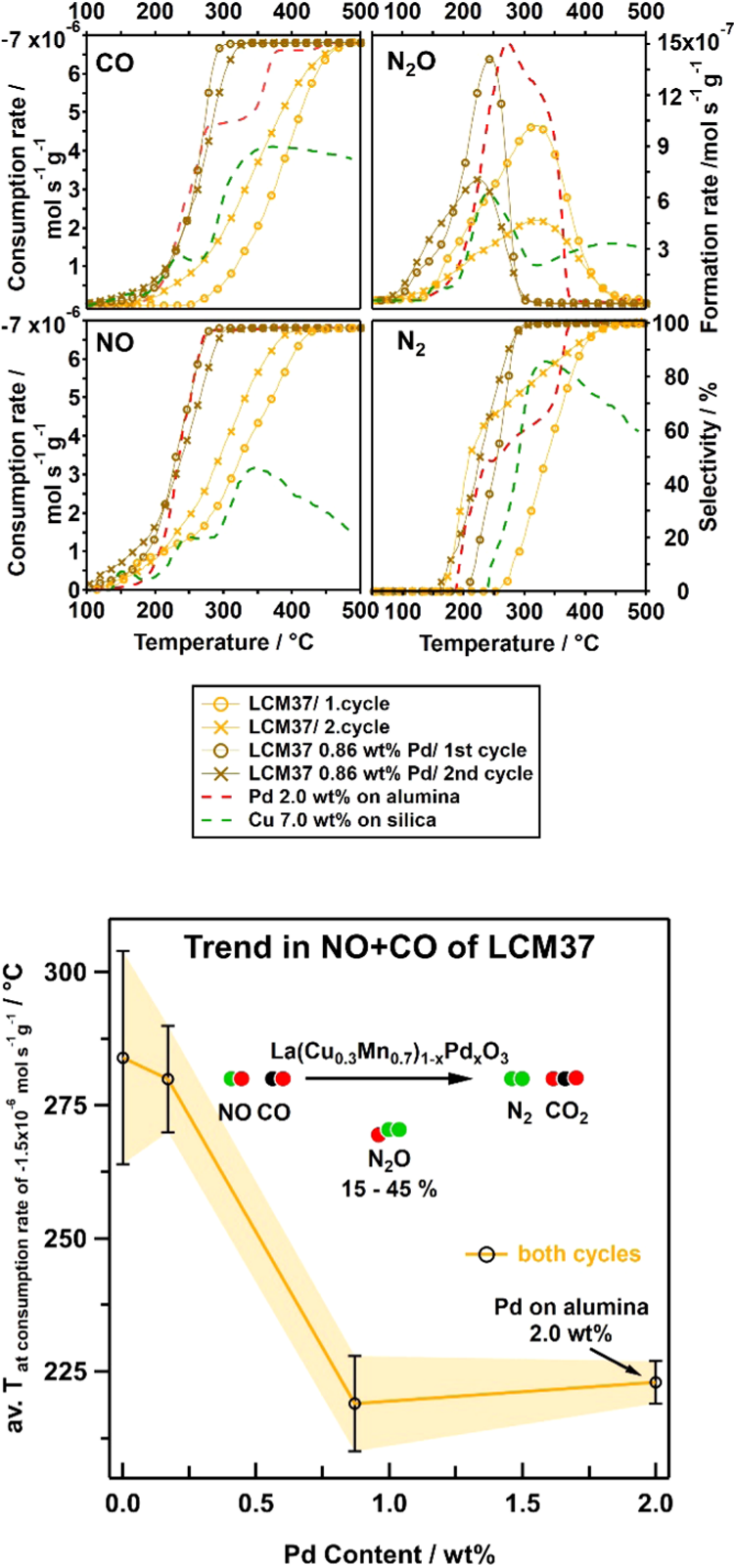
Top panel: consumption-rate profiles of CO and NO, intermediate formation-rate profiles of N_2_O, and respective N_2_-selectivity profiles illustrate the catalytic performances of pure LCM37 (orange, circles first and crosses second cycle) and 0.86 wt % Pd-doped LCM37 (brown, circles first and crosses second cycle) in comparison to the reference catalysts Pd-alumina (red dashed line, 1 cycle) and Cu-silica (dark green dashed line, 1 cycle). Bottom panel: average of the temperatures for a consumption rate of −1.5 × 10^–6^ mol s^–1^ g^–1^ of NO and CO of both cycles, illustrating the activity trend of the LCM37 system depending on the Pd content.

The general trend in the catalysis of the LCM37-based catalysts is illustrated in Figure 4, bottom panel (see Supporting Information Appendix B, Table S3 for detailed numerical values) and shows a clear dependence of the temperatures required for a certain consumption rate on the Pd content. The resulting catalyst’s performances were plotted as the consumption rate of CO and NO, intermediate formation rate of N_2_O, and N_2_ selectivity vs the temperature (Figure 4, top panel) and were further related to two reference catalysts (2.0 wt % Pd on alumina and 7.0 wt % Cu on silica), depicted as red and green dashed lines, respectively. For more details concerning the reference catalyst’s characterization, see Supporting Information Appendix I, Figure S15. For reasons of clarity, the catalytic behavior of the samples with low Pd content (0.18 wt %) is not illustrated in the top panel.

Already, the 0.86 wt % Pd-doped LCM37 sample (brown circles and crosses) features a comparable activity in NO and CO conversion as the 2.0 wt % Pd reference catalyst and even outperforms it in terms of nitrogen selectivity (100% below 300 °C). The increased nitrogen selectivity can be directly correlated to the improved N_2_O conversion rate at lower temperatures. The stagnation of the CO consumption rate between ∼270 and 350 °C on the Pd reference catalyst (pronounced shoulder in the upper left panel of Figure 4, corresponding to the broad region with a high formation rate of intermediate N_2_O in the upper right panel), is most likely due to a delayed onset of reaction 2 by intermediate Pd poisoning by CO_ads_, i.e., by blocking of metallic reaction sites for N_2_O decomposition. As known from the work by Dropsch and Baerns, the thermal CO desorption from Pd becomes increasingly fast at around 340 °C.^61^ We, therefore, assume that the onset of CO desorption leads to an increase of Pd surface-adsorption sites for N_2_O and—therefore—to a strongly accelerated N_2_O decomposition above this temperature. The absence of this CO-stagnation phenomenon on the Pd-doped LCM37 catalyst (0.86 wt %) is attributed to a combination of available active Pd- and Cu-LCM interfacial sites associated with simultaneous reduction of CO poisoning of the metallic surface species. Alloying of Pd by Cu (Figure 3) most likely alters Pd sites electronically, which might explain the slight decrease of the conversion temperatures for NO and CO in the second cycle. Nevertheless, the Pd-doped LCM sample exhibits a superior performance both in activity and selectivity. Additional exsolution of Cu can explain the generally observed decrease of the amount of intermediate N_2_O in the respective second catalytic cycles (Figure 4, top panel, crosses vs cycles), both on pure and Pd-doped LCM37, as Cu is even less prone to CO poisoning.^62,63^ This trend is particularly obvious in the case of pure LCM37 (orange) on which Cu exsolution causes strongly improved N_2_O decomposition chemistry and overall activity observed in the second catalytic cycle. Cu(0) is well known as an efficient catalyst for N_2_O decomposition^50,51^ and is thus held responsible for this phenomenon. To prove if either the metallic Cu surface itself, or the interface Cu(0)/perovskite provides the active sites for the reaction on LCM37, we compared the results with an interface-inactive reference catalyst: Cu on inert silica (Figure 4). Before starting the first catalytic cycle, the metallic state of Cu was ensured by a reductive pretreatment of the catalyst (3 h, 350 °C, 20% H_2_ in He).

Although Cu/SiO_2_ can poorly activate NO under intermediate formation of N_2_O in the first cycle, this reaction pathway is largely suppressed in the second cycle and only some residual high-temperature consumption of NO at temperatures *T* > 300 °C is observed (see Supporting Information Appendix I, Figures S15 and S16). This oxidative deactivation of Cu/SiO_2_ is absent in the Cu(0)/perovskite-based catalysts and provides a first indication of distinct interfacial chemistry. The first conclusion from the generally rather low activity and subsequent deactivation of Cu/SiO_2_ leading to a high-temperature shift of NO conversion (>200 °C in first vs >300 °C in the second cycle) is that the interfacial bifunctionality of metallic Cu and the perovskite is fundamental for the more efficient NO and CO conversion on the LCM37 catalyst, which rather tends toward self-activation. By comparison with the Cu-SiO_2_ reference catalyst, which is only capable of efficient N_2_O conversion in the first cycle (a fast drop of N_2_O at temperatures above 200 °C, followed by rapid deactivation), we infer that the combination of enhanced N_2_ selectivity at lower temperatures and the strong promotion of the overall NO + CO reaction results from the interaction of a stabilized metallic Cu surface with the beneficial role of the Cu-LCM phase boundary. Fast N_2_O degradation is an intrinsic property of metallic Cu,^50,51^ whereas efficient and reproducible NO activation appears to be linked to the presence of redox-active phase-boundary sites.

**Figure 5:**
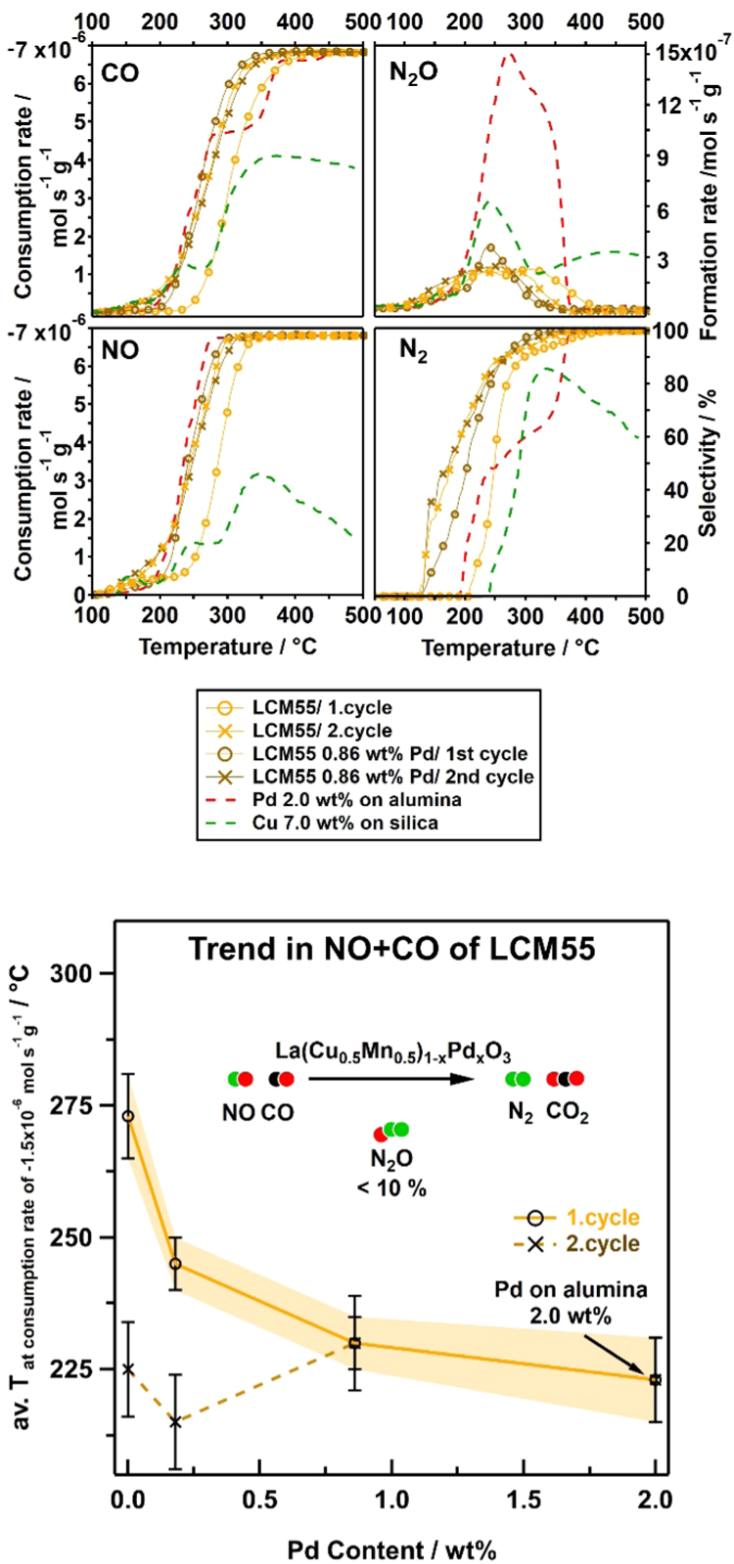
Top panel: consumption-rate profiles of CO and NO, intermediate formation-rate profiles of N_2_O and respective N_2_-selectivity profiles illustrate the catalytic performances of pure LCM55 (orange, circles first and crosses second cycle) and 0.86 wt % Pd-doped LCM55 (brown, circles first and crosses second cycle) in comparison to the reference catalysts Pd-alumina (red dashed line, 1 cycle) and Cu-silica (dark green dashed line, 1 cycle). Bottom panel: average of the temperatures for a consumption rate of −1.5 × 10^–6^ mol s^–1^ g^–1^ of NO and CO of each cycle, illustrating the first and second cycle activity trend of the LCM55 system depending on the Pd content.

The catalytic activity and selectivity plots for the LCM55 samples are accordingly shown in Figure 5, top panel. The measurements are again compared with the reference catalysts (2.0 wt % Pd on alumina and 7.0 wt % Cu on silica), indicated by red and green dashed lines in each plot. Both undoped and Pd-doped LCM55 exhibit a much higher selectivity toward nitrogen at lower temperatures as compared to the reference catalysts. The general trends of the activities and selectivities on the LCM55 catalysts are entirely different from those observed on LCM37 catalysts (Figure 5 vs Figure 4, bottom panels, see Supporting Information Appendix B, Table S4 for detailed numerical values). A much weaker dependence of the consumption-rate temperatures on the Pd content is evident already in the first cycle, and the promotional Pd effect becomes practically overruled in the second catalytic cycle. The generally higher activity of pure LCM55, as compared to pure LCM37, most likely stems from the combination of a generally increased Cu(0) exsolution and an intrinsically higher number of oxygen vacancies due to the higher lattice-Cu loading, leading to an enhanced density of active interfacial sites. It is known from Petrov et al.^47^ that higher Cu loadings in LCM-based materials decrease the lattice cell volume and thus cause a reflex shift in PXRD patterns toward higher angles. Additional oxygen vacancies just compensate for this trend in both undoped and doped LCM55 (Supporting Information Appendix J, Figures S17 and S18 and Tables S9 and S10). Nevertheless, a reflex shift toward lower angles was observed in all catalysts after the NO + CO reaction, indicating a lattice expansion in terms of additional oxygen-vacancy formation. This effect is slightly more pronounced on pure LCM55, indicating a more pronounced defect chemistry. Most likely, the increased oxygen affinity of the pure LCM55 catalyst allows decomposing NO much more effectively,^18^ and the enhanced amount of Cu nanoparticles provides immediate degradation of intermediate N_2_O toward N_2_.

The minor parasitic La_2_CuO_4_ cuprate phase was found to be catalytically considerably less active than the perovskite LCM catalysts^64^ and can therefore be excluded to contribute in the catalytic performance substantially (Supporting Information Appendix K, Figure S19).

To establish the effect of Cu nanoparticle aggregation on the catalytic performance, a long-term experiment for the most active Pd-free catalyst LCM55 was conducted (Supporting Information Appendix L, Figure S20) and revealed only a minor decrease in the CO_2_ formation during 96 h time-on-stream at a temperature where initially 100% CO_2_ formation was observed.

### 3.3 Correlation of Surface Composition to NO + CO Reactivity

**Figure 6:**
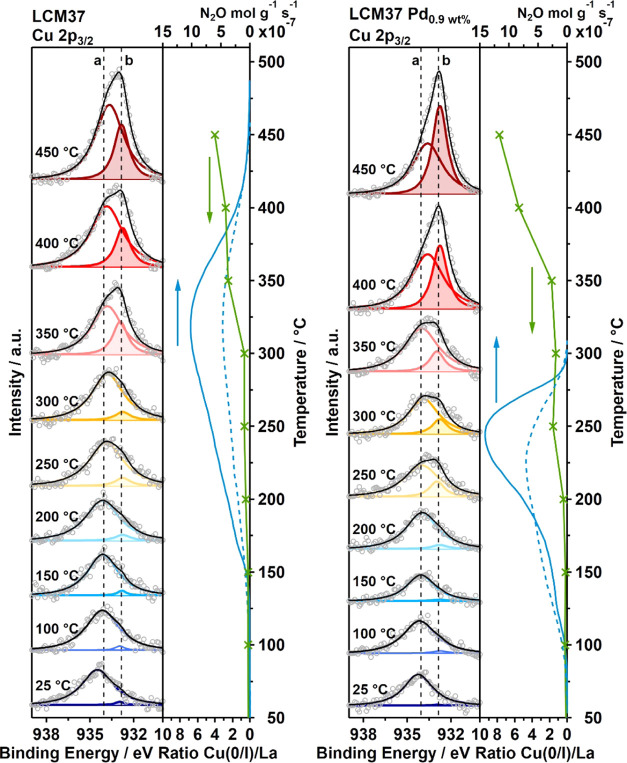
Cu 2p_3/2_ XP spectra recorded *in situ* under CO + NO atmosphere (0.3 mbar each) for nine isothermal temperature steps between 25 and 450 °C for pure LCM37 (left panel) and 0.86 wt % Pd-doped LCM37 (right panel). Experimental data: gray circles; colored fits components: unshaded Cu(II) and shaded Cu(0); vertical dashed lines: BE reference values for Cu(II) 934.06 eV and Cu(0) 932.86 eV, see Supporting Information Appendix M, Figure S22; fit envelope: black line. On the right, the La 3d_5/2_-normalized integral intensity of Cu(O) (green line with crosses, determined from the color-shaded peak fittings) is directly compared to the formation rate of intermediate N_2_O (blue solid line: first cycle, blue dashed line: second cycle), as taken from the catalytic measurements.

To elucidate the surface-oxidation state of nanoparticular exsolved Cu and CuPd on LCM, we carried out *in situ* NAP-XPS measurements on powder-based catalysts under a controlled NO + CO atmosphere. In general, the comparison of the recorded Cu 2p_3/2_ and Cu LMM *in situ* XP spectra with reference substances (metallic Cu, Cu_2_O, and CuO) enables us to quantify changes in the Cu-oxidation state depending on the applied temperature and gas phase. To directly compare the temperature-dependent oxidation states of Cu in pure and 0.86 wt % Pd-doped LCM37 and LCM55, all recorded spectra are waterfall-plotted with increasing temperature steps from bottom to top, presented in Figures 6 and 8 for each catalyst sequentially. By fitting the Cu 2p_3/2_ region with two components (GL90 lineshape) representing Cu(II) (934.06 eV) and Cu(0)/Cu(I) (932.86 eV) and normalizing to the La 3d_5/2_ peak area, the ratios (Cu/La) of these two distinguishable Cu-oxidation states can be determined. As the La content is the same in all samples, fitting of the Cu 2p_3/2_ region with two components representing Cu(II) and Cu(0)/Cu(I) and normalization of the respective integral intensities to the La 3d_5/2_ peak area allows us to quantify both the relative and total surface-near contributions of those two distinguishable Cu-oxidation states (for details concerning the fitting procedure, normalized values, and reference data, see Supporting Information Appendix M, Figures S21 and S22 and Tables S11 and S12).

By comparing the Cu LMM Auger spectra with the references derived from sputter-cleaned metallic Cu, Cu_2_O, and CuO, a further discrimination between Cu(0) and Cu(I) species can be established. This allows us to exclude participation of a Cu(I) species to the catalytic process at least for the Pd-containing catalysts and pure LCM37 (Supporting Information Appendix M, Figure S21). In contrast, pure LCM55 shows a pronounced Cu(I) Auger signal between 400 and 475 °C (Figure 9).

The trend of the Cu(0) contribution upon heating in 0.6 mbar NO + CO is in a very good agreement with the catalytic data, especially with respect to the N_2_O chemistry on the catalysts. To illustrate how the interfacial Cu(0)/LCM contribution affects the behavior of intermediate N_2_O, Figures 6 and 8 combine catalytic data with spectroscopic measurements in elucidating the dependence of the N_2_O decay on the availability of Cu(0)/LCM reaction sites. Note that the scale of the N_2_O formation-rate axis and the ratio of Cu(0) + Cu(I) to La 3d_5/2_ axis in Figures 6 and 8 is adjusted to directionally highlight the correlation of the surface chemistry to the catalytic performance.

Figure 6 corroborates the catalytic results for pure LCM37, which clearly show a maximum of the N_2_O formation rate at about 320 °C, the ratio Cu(0)/La in the Cu 2p_3/2_ spectra between 300 and 350 °C increases from 0.6 to 2.5. The same applies for Pd-doped LCM37 on which the N_2_O formation-rate maximum at 240 °C perfectly matches the increasing Cu(0) contribution within the same temperature range (between 200 and 250 °C) from 0.4 to 1.6. We conclude that Cu(0) enrichment of the surface leads in any case to a more effective conversion of N_2_O toward N_2_.

**Figure 7:**
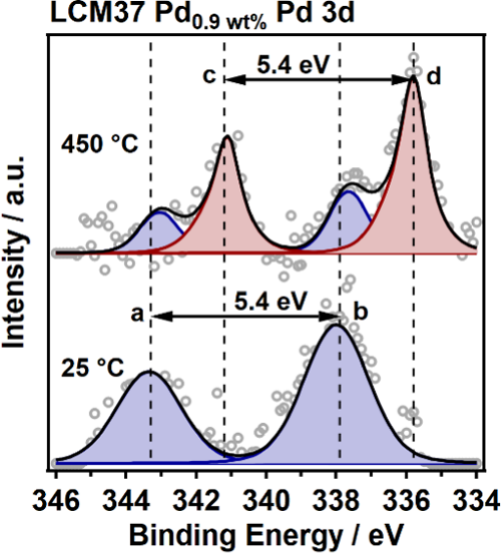
Pd 3d XP spectra recorded *in situ* under a CO + NO atmosphere (0.3 mbar each) for two isothermal temperature steps at 25 and 450 °C for 0.86 wt % Pd-doped LCM37. Experimental data: gray circles; blue shaded fit components: oxidized Pd(IV); red shaded fit components: metallic/alloyed Pd(0). Vertical dashed lines: BE reference values for (a) Pd(IV) 3d_3/2_ 343.3 eV, (b) Pd(IV) 3d_5/2_ 337.9 eV, (c) Pd(0) 3d_3/2_ 341.2 eV, and (d) Pd(0) 3d_5/2_ 335.8 eV.^65−67^ Resulting fit envelope: black line.

In general, the ratios of Cu(0)/La for LCM37 range from 0.2 at 25 °C to 4.1 at 450 °C in contrast to the Pd-doped analogue starting at a ratio of 0.1 at 25 °C, but ending with a much higher value of 7.8 at 450 °C. The pronounced increase of the Cu(0) contribution in Pd-doped LCM37 above 350 °C agrees with the Pd-assisted exsolution observed via the microscopic investigations (cf. Figure 3). Beyond this result, the exsolution-enhancing role of Pd was directly validated by measuring *in situ* XP spectra of the Pd 3d region of Pd-doped LCM37 at room temperature and 450 °C (Figure 7). Keeping up a fixed doublet splitting (3d_5/2_ to 3d_3/2_ of 5.4 eV), the highly oxidized Pd 3d region (blue shaded peaks) at 25 °C shifts toward metallic/alloyed Pd species at 450 °C. The binding energy (BE) of 337.9 eV of the 3d_5/2_ peak marked with (b) in Figure 7 is very similar to the BE found for PdO_2_; thus, a Pd(IV) valence state can be formally assigned.^65,66^ The 3d_5/2_ peak marked with (d) at BE of 335.8 eV, recorded at 450 °C, can be attributed to Pd(0). Moreover, the respective peak splitting of 5.4 eV is in very good agreement with the literature values for Pd and PdO.^65,67^

**Figure 8:**
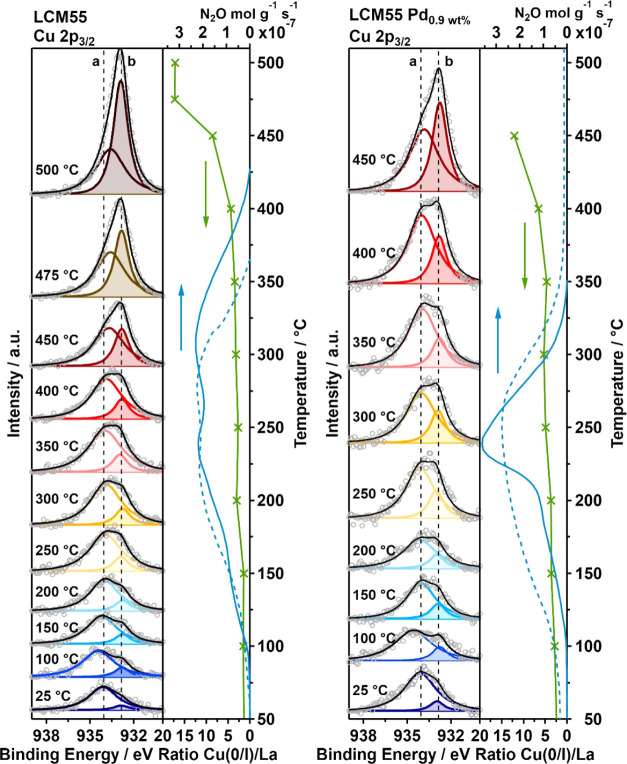
Cu 2p_3/2_ XP spectra recorded *in situ* under CO + NO atmosphere (0.3 mbar each) for nine resp. eleven isothermal temperature steps between 25 and 450 °C resp. 500 °C for pure LCM55 (left panel) and 0.86 wt % Pd-doped LCM55 (right panel). Experimental data: gray circles; colored fit components: unshaded Cu(II) and shaded Cu(0); vertical dashed lines: BE reference values for Cu(II) 934.06 eV and Cu(0) 932.86 eV, see Supporting Information Appendix M, Figure S22; fit envelope: black line. On the right-hand vertical plot side, the La 3d_5/2_-normalized integral intensity of Cu(O) (green line with crosses, determined from the color-shaded peak fittings) is directly compared to the formation rate of intermediate N_2_O (blue solid line: first cycle, blue dashed line: second cycle), as taken from the catalytic measurements.

In accordance with the XRD results showing Cu(0) in pure and Pd-doped LCM55 already after synthesis, we detected some Cu(0) in the Cu 2p_3/2_ region already at 25 °C (Figure 8) (ratio of Cu(0)/La in pure LCM55: 1.3 and in Pd-doped LCM55: 2.1). During the heating of pure LCM55, we observe a slowly increasing amount of Cu(0)/Cu(I) with increasing temperature until 350 °C and more pronounced above 400 °C. As soon as the temperature reaches 310 °C, N_2_O starts to decompose similarly, but at slightly lower temperatures compared to the pure LCM37 catalyst. However, the N_2_O formation rate is lower by a factor of 4.5 for LCM55 compared to LCM37 during the first cycle, directly indicating the activity of the already present Cu(0)/LCM interface. The temperature dependence of the N_2_O decay on Pd-doped LCM55 (right panel in Figure 8) indicates that the catalyst behaves similarly as Pd-doped LCM37, i.e., both start to decompose N_2_O at 240 °C. In contrast to Pd-doped LCM37 (Figure 6), the N_2_O formation rate of Pd-containing LCM55 (Figure 8) is reduced by a factor of 4.5 (see different axis scale in the respective figure), corroborating the line of argumentation of the importance of sufficient metallic CuPd surface and/or vacancy-doped CuPd/LCM interfacial sites with respect to N_2_O decomposition.

**Figure 9:**
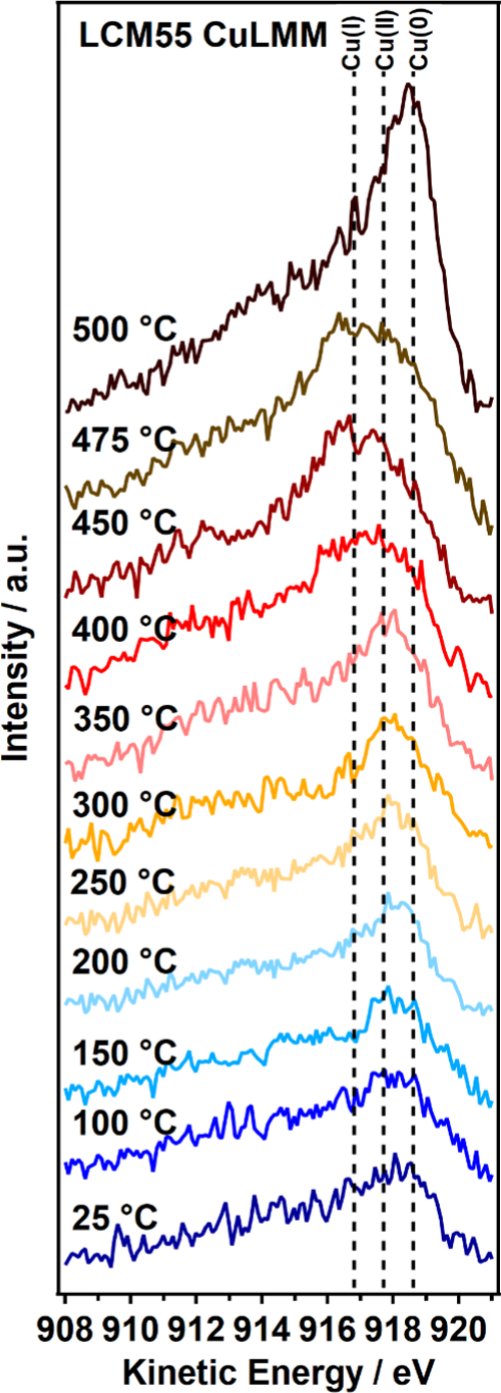
X-ray excited *in situ* Auger spectra recorded under CO + NO atmosphere (0.3 mbar each) in the NAP-XPS instrument for 11 isothermal temperature steps between 25 and 500 °C. The Cu LMM auger region was recorded for pure LCM55 (experimental data: colored solid lines; vertical dashed lines: BE reference values for Cu(I) 916.8 eV, Cu(II) 917.7 eV, and Cu(0) 918.6 eV; see Supporting Information Appendix M, Figure S22).

By comparing the distinct trends of Cu(0) surface enrichment, it turns out that for the Pd-containing catalysts, this process is always enhanced. Aside from the promoting effect of metallic Pd, which appears to both induce and stabilize Cu(0) nucleation right from the beginning, the exsolution chemistry of Cu(0) seems to be more facilitated in LCM37 than in pure LCM55, the latter showing a considerably stronger Cu(I) contribution at elevated temperatures. To prove and subsequently overcome a possible hindered nucleation procedure for Cu(0) on pure LCM55, we extended the heating to 500 °C, in consistency with the *ex situ* catalytic flow reactor measurements in the NO + CO atmosphere. By directionally mimicking the *ex situ* treatment of LCM55 in the NAP-XPS experiment, we could identify a strongly increasing Cu(I) contribution until 475 °C (Figure 9, red to brown trace), followed by an even more pronounced Cu(0) growth (Cu(0)/La ratio approaching 17.2). This final value is comparable with the intensity found in the Pd-containing catalyst, as soon as 500 °C is reached (Figures 8 and 9, dark brown traces). Taken together, these findings in essence can be directly correlated to the strongly enhanced activity of pure LCM55 in the second catalytic cycle (Figure 5). Once a comparable amount of exsolved Cu(0) is reached, which requires the decisive step to 500 °C, LCM55 exhibits similar catalytic properties as the Pd-containing LCM55-based material and the 2.0 wt % Pd/alumina reference catalyst, respectively. In analogy to CuPd, we interpret these results in terms of sufficient metallic Cu surface and/or vacancy-doped Cu/LCM interfacial sites, promoting efficient low-temperature N_2_O decomposition.

To determine how the amount of metallic Cu evolves in a second catalytic cycle, a sequential in situ XP experiment was carried out. During the second catalytic cycle, the oxidation state of Cu shifts from Cu(0/I) to Cu(II). In the catalytically active temperature region between 200 and 300 °C, we detect an area of a stable Cu(II)/Cu(0/I) ratio manifesting itself as the active state of Cu. Conclusively, this result strongly supports our proposed reaction mechanism, showing an intermediate partial oxidation of metallic Cu during the conversion of NO and CO. By further increasing the temperature to 500 °C, a significantly higher amount of Cu(0/I) was observed. We suggest that higher temperatures enhance both the Cu mobilities, resulting in a more pronounced Cu-particle growth, and the enhancement of the reaction kinetics suppresses the detection of the intermediate oxidation of metallic Cu sites (Supporting Information Appendix N, Figure S23).

## 4 Conclusions

**Figure 10:**
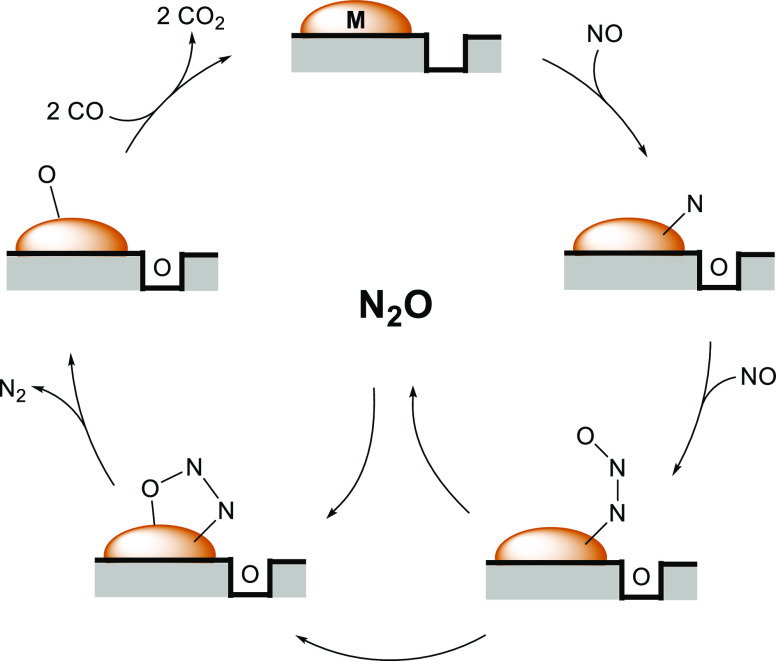
Reaction mechanism on LCM-based catalysts, proposing a metal (M) to oxygen-deficient perovskite interface acting as the active site for NO dissociation, intermediate N_2_O formation, and CO-induced regeneration.

With respect to the impact of the *in situ* formed Cu(0) surface/perovskite interfacial sites on the NO + CO reaction pathway, we demonstrate that the Cu(0)/perovskite interface undergoes a catalytic redox cycle of oxygen-vacancy quenching by NO dissociation and subsequent vacancy reformation by CO oxidation (Figure 10). As one of the most fundamental outcomes of our work, the self-activation behavior of the pure LCM55 catalyst under the NO + CO reaction conditions implies a similar, but more Cu(0)-based bifunctionality as on the Pd-doped LCM37 systems: Cu(0) can gradually or even fully substitute Pd(0) mechanistically, at least under the chosen reaction conditions. This is only possible on the pure LCM55 catalyst due to the enhanced high-temperature Cu(0) exsolution (Figure 8) and vacancy chemistry.^18^ In due course, this leads to the surprising situation that even the Pd-free LCM55 catalyst becomes similarly active and selective as the doped 0.86 wt % Pd-containing LCM37 catalyst (cf. Figure 4). On LCM37, the catalytic promotion by Pd cannot be fully replaced by pure Cu(0). A possible explanation can be offered in the context of Figure 3, showing the formation of mostly alloyed Cu-rich Cu/Pd particles on Pd-doped LCM37. The identical-location EDXS experiment of the particle area provides evidence for a dilute alloy state of Pd in Cu(0), already approaching a pure Cu(0) particle state. In terms of self-activation, further increase in activity of pure LCM55 in the second cycle is most likely due to the strongly increased number of active centers at additional Cu(0)/LCM55 interfaces (cf., Figure 8 and Supporting Information Appendix C, Figure S4, Appendix J, Figures S17 and S18, and Appendix N, Figure S23). As a general consequence, sufficiently Cu-rich LCM systems are proper noble metal-free catalysts as they provide a sufficient density of active interfaces in the reduction of NO by CO and allow us to replace noble metals (e.g., Pd) at least with respect to the specific NO + CO pathway.

## Supporting Information Available

Data evaluation of catalytic tests; XRD characterization of LCM37 samples; determination of Cu(0) + Cu(I) vs Cu(II) from XPS data with a peak-fitting model according to Biesinger et al.; structure and morphology characterization of LCM37 with and without Pd by TEM; structure and morphology characterization of LCM55 with and without Pd by TEM; XPS measurements of pure and Pd-doped LCM55 catalysts, with the respective fitting procedure to evaluate the amount of Cu(0) + Cu(I) vs Cu(II); BET specific surface area measurements of undoped and Pd-doped LCM-based catalysts; XPS investigation of the Cu/SiO_2_ reference catalyst; reflex shift data evaluation for undoped and Pd-doped LCM-based catalysts; catalytic characterization of La_2_CuO_4_; *in situ* XP spectra of the Cu LMM auger region, Cu reference material investigation, and data evaluation of *in situ* XP spectra of the LCM-based Cu 2p_3/2_ region; kinetic studies and particle coverage estimation of LCM-based catalysts; further NAP-XPS measurement to monitor the Cu state during the second catalytic cycle; and long-term measurement of LCM55 (PDF)
