# Supplementary material for: Who Does the Job? How Copper Can Replace Noble Metals in Sustainable Catalysis by the Formation of Copper–Mixed Oxide Interfaces
Source: ACS Catal. 2022 Jun 14;12(13):7696–708. doi: 10.1021/acscatal.2c01584 (PMC9251726; doi:10.1021/acscatal.2c01584)
Supplement: Supplementary file 1 — cs2c01584_si_001.pdf [file cs2c01584_si_001.pdf]

Supporting Information

Who does the job? How copper can replace noble metals in sustainable catalysis by formation of copper-mixed oxide interfaces

Christoph W. Thurner,[1] Nicolas Bonmassar,[2] Daniel Winkler,[1] Leander Haug,[1] Kevin Ploner,[1] Parastoo Delir Kheyrollahi Nezhad,[1,3] Xaver Drexler,[1] Asghar Mohammadi,[1,3] Peter A. van Aken,[2] Julia Kunze-Liebhäuser,[1] Aligholi Niaei[3], Johannes Bernardi,[4] Bernhard Klötzer[1] and Simon Penner[1,*]

[1]*Department of Physical Chemistry, University of Innsbruck, Innrain 52c, A-6020 Innbruck, Austria*

[2]*Max Plank Institute for Solid State Research, Heisenbergstaße 1, D- 70569, Stuttgart, Germany*

[3]*Reactor & Catalyst Research Lab, Department of Chemical Engineering, University of Tabriz, 29 Bahman Blvd, Tabriz, Iran*

[4]*Technische Universität Wien, USTEM / 057-02, Wiedner Hauptstr. 8-10, A-1040 Wien*

Keywords: CO • copper • exsolution • interface • metal-mixed oxide • NO abatement • N_2O • palladium • perovskite

*Corresponding author: S. Penner, simon.penner@uibk.ac.at

APPENDIX A: Kinetic studies and particles coverage estimation of LCM-based catatlysts.

To determine the activation energy (E_a) and pre-exponential factor we fitted the catalytic data (i.e. initial consumption rate at low temperatures) with an Arrhenius fit function $A \cdot \exp(-E_a/(R \cdot T))$. Figure S1 illustrates the fit of the consumption rate data of pure LCM55 (blue line) and Pd-doped LCM55 (red line) for CO and NO respectively. In addition, the fit was carried out for the first (solid line) and the second (dashes line) cycle.

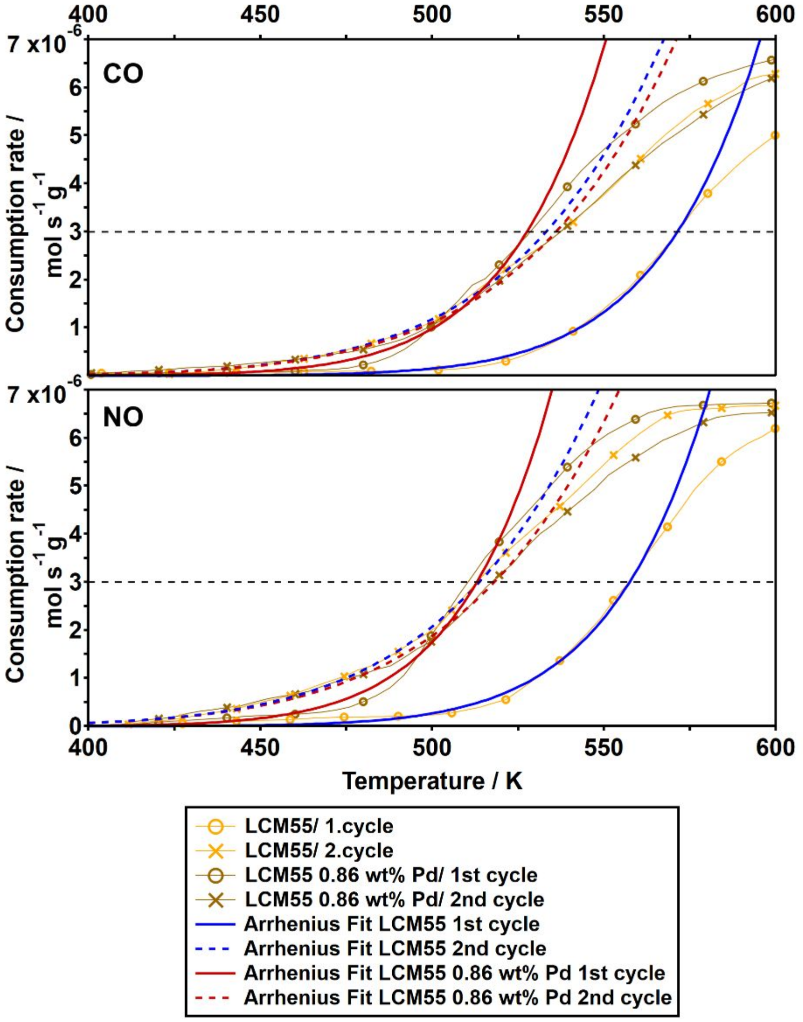

Figure S1. Fits of the onset region of the consumption rate traces of CO and NO for pure LCM55 (blue lines) and Pd-doped 0.86 wt% LCM55 (red lines). Fits of the first catalytic cycle are plotted with solid lines, whereas fits of the second catalytic cycle are illustrated as dashed lines.

To suppress rate-influencing effects originating from the decreasing concentration of educts the fit was constrained to sufficiently small initial changes of the reactant pressures, corresponding to the temperature region up to a consumption rate of $3 \cdot 10^{-6}$ mol s^{-1} g^{-1} (dashed line in Figure S1). The fit parameters are summarized in Table S1.

Table S1. Fit parameters derived from the fits of the CO and NO consumption rates traces illustrated in Figure S1 given for pure and Pd-doped LCM55.

Catalyst	Cycle	Pre-Exponential Factor A / mol s^{-1} g^{-1}	Error u_A / mol $s^{-1}g^{-1}$	Activation Energy E_a / kJ	Error u_E / kJ
			CO		
**LCM55**	1	$4 \cdot 10^3$	$\pm 1 \cdot 10^3$	99	± 2
	2	3.9	± 1.8	62	± 2
**LCM55 Pd 0.86 wt%**	1	$2 \cdot 10^3$	$\pm 1 \cdot 10^3$	89	± 3
	2	3.1	± 0.6	62	± 1
			NO		
**LCM55**	1	$4 \cdot 10^3$	$\pm 2 \cdot 10^3$	98	± 2
	2	2.0	± 0.6	57	± 1
**LCM55 Pd 0.86 wt%**	1	$3 \cdot 10^3$	$\pm 2 \cdot 10^3$	88	± 3
	2	1.2	± 0.8	56	± 3

The following interpretation is limited to a qualitative statement only and should give a rough but representative trend. The activation energy drops from the first to the second catalytic cycle from ~95 kJ to ~60 kJ meaning that the evolving Cu/LCM55 interface lowers the activation barrier. In addition, the drop the of pre-exponential factor from $\sim 10^3$ to $\sim 10^0$ can be interpreted in terms of a decrease in the total amount of reaction sites. Therefore, we suggest a high amount of less active perovskite surface sites in the first cycle vs. a lower amount of kinetically promoted interfacial Cu/LCM55 sites becoming dominant in the second catalytic cycle. The change of the first vs. second cycle pre-exponential factor is about 10^3 which agrees with the most recently estimated ratio of surface sites of the bare perovskite vs. interfacial Cu/LCM sites (see below).

To calculate the surface coverage of Cu and Cu_y/Pd_x particles we aimed on the the La3d and Cu2p NAP-XPS intensity at 450 °C to overcome eventual limitations of the local TEM information. At this temperature, sufficiently large and well-grown particles are already present on the surface. The utilization of an attenuated particle coverage model based on work of C.S. Fadley[1] enables us to determine an average Cu particle coverage. Upon consideration of half-spherical particle shapes and assuming an averaged particle size of 10 nm derived from the HRTEM results, a particle coverage (i.e., particle density per surface area) was calculated. Table S2 gives a comparison of the obtained coverages for all LCM-based catalyst materials.

Table S2. Calculated surface coverage values for Cu nanoparticles on LCM-based catalyst compositions.

Catalyst	LCM37	LCM37 Pd 0.86 wt%	LCM55	LCM55 Pd 0.86 wt%
**Cu-covered surface fraction**	0.010	0.019	0.021	0.030

The Pd-containing catalysts show even higher particle coverages compared to the Pd-free catalysts. Therefore, we conclude that sufficient Cu_yPd_x/perovskite interface is present to participate to the catalytic performance.

The equation used to determine the particle coverage x is given as:

$$\frac{N_p(\theta)}{N_s(\theta)} = \frac{x \cdot I_p \cdot \rho_p \cdot \frac{d\sigma_p}{d\Omega} \cdot \Lambda_p(E_p) \cdot cos\theta \cdot \left(1 - exp\left(\frac{-t}{\Lambda_p(E_p) \cdot cos\theta}\right)\right)}{x \cdot I_s \cdot \rho_s \cdot \frac{d\sigma_s}{d\Omega} \cdot \Lambda_s(E_s) \cdot cos\theta \cdot \left(exp\left(\frac{-t}{\Lambda_p(E_s) \cdot cos\theta}\right)\right) + (1-x) \cdot I_s \cdot \rho_s \cdot \frac{d\sigma_s}{d\Omega} \cdot \Lambda_s(E_s) \cdot cos\theta}$$

ρ … Atom density / cm^{-3}

I … X-ray flux (constant)

$\frac{d\sigma}{d\Omega}$… differential cross section (data SRD 64 data base[2])

θ … analysis angle

t … effective particle thickness (cylindrically idealized particles derived from half-spherical shape)

N … normalized XPS Intensity (peak area)

$\Lambda(E)$ … electron attenuation length (data from SRD 82 data base[3])

x … particle coverage

Indices: p... particle; s… substrate

We note that an average value for the parameter t has been derived from a series of TEM particle shapes, so the only remaining parameter x can be calculated from the La3d/Cu2p intensity ratios. From the particle coverage calculated, interfacial Cu/LCM sites can be geometrically estimated (half-sphere particle shape) and compared with the total number of surface sites (assumed to correspond to the number of oxide unit cells per surface area) of the bare perovskite. The ratio of surface to interfacial sites is in the range of 10^3 which agrees with the kinetic studies.

APPENDIX B: Data evaluation of the catalytic tests

The MS signals for the current NO concentration (S_C), for the initial flow of 10000 ppm NO (S_I) and for the He blank measurement (S_0) were used to determine the consumption of NO in the gas flow X_{NO} given in a more general way as X_{gas} with a value between 0 and 1 see Eq. (1)

$$X_{gas} = 1 - \frac{S_C - S_0}{S_I - S_0}, [0,1] \tag{1}$$

Further the ideal gas equation, the contact time $\tau = 1/\mathrm{GHSV} = V_{gas}/\dot{V}$, the catalyst mass $m_{cat} = 0.200$ g and the flowrate $\dot{V} = 200\ \mathrm{ml/min}$ were used to calculate the mass specific consumption rate $A_{c,ms,gas}/\mathrm{mol/s \cdot g}$ see Eq. (2).

$$-\frac{\mathrm{d}c_{gas}}{\mathrm{d}t} = A_{c,ms,gas} = \frac{p \cdot V_{gas}}{R \cdot T} \cdot \frac{X_{gas}}{m_{cat} \cdot \tau} = \frac{p \cdot \dot{V} \cdot X_{gas}}{R \cdot T \cdot m_{cat}} \tag{2}$$

For the temperature and the pressure, the following conditions were used $p = 101325\ \mathrm{Pa}$ and $T = 298{,}15\ \mathrm{K}$ (ideal gas constant $R = 8.314\ \mathrm{J \cdot mol^{-1} \cdot K^{-1}}$). With the approach of equation (1) and (2), but instead using the IR absorbance at 2115 cm^{-1} wavenumbers, the CO conversion and in the following the mass specific consumption rate of CO was calculated. As CO_2 (gas phase IR absorbance at 2360 cm^{-1}) exhibited a response to partial pressure changes deviating from Lambert-Beer's law, a calculation of CO_2 formation was not carried out. The superposition of the QMS signals of carbon dioxide and nitrous oxide (both 44 m/z) was resolved with the help of the IR-setup. An external calibration of N_2O in He (Figure S2), especially the slope (8746 ppm) of the absorbance at 2237 cm^{-1} wavenumbers (anti-symmetric stretching vibration) was used to determine the current concentration of intermediate N_2O ($S_{c,N2O} = \log\left(I_0/I\right) \cdot 8746$). The determination of the formation of N_2O (Y_{N2O}, Intervall [0,1]) was further done by Eq. (3) using the maximum concentration ($S_{m,N2O}$) of 5000 ppm, under consideration of the stoichiometry (NO:N_2O = 2:1)

$$Y_{N2O} = \frac{S_{c,N2O}}{S_{m,N2O}}, [0,1] \tag{3}$$

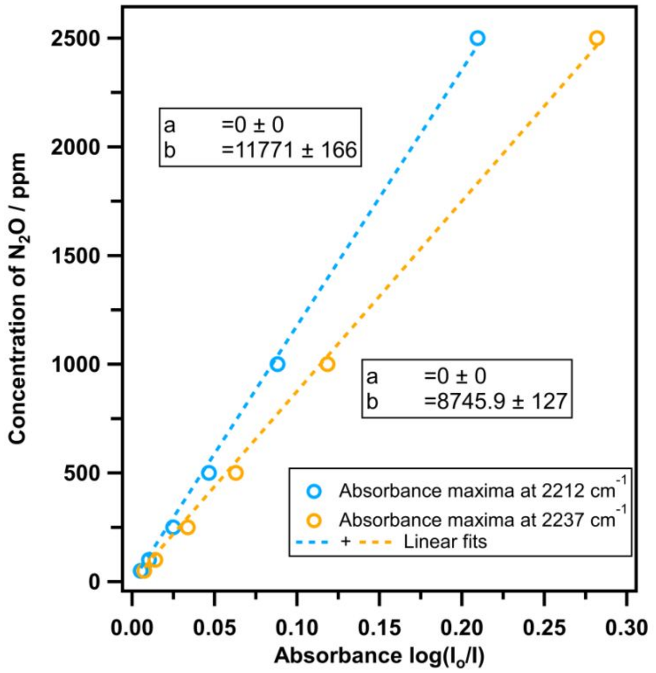

Figure S2. External standard calibration of the IR-setup with N_2O in helium (50, 100, 250, 500, 1000 and 2500 ppm). The circles describe the absorbance maxima at 2212 cm^{-1} (blue circles) and at 2237 cm^{-1} (orange circles) and the dashed lines the respective linear fit.

The calculation of the formation rate of N_2O ($A_{f,\mathrm{ms,N20}}/{}^{mol}/_{s \cdot g}$) was done in a very similar way to Eq. (2), but additionally using 0.5 as the factor to correct for the maximum concentration of N_2O (5000 ppm).

$$\frac{dc_{N20}}{dt} = A_{f,\mathrm{ms,N20}} = \frac{p \cdot \dot{V} \cdot Y_{N20}}{R \cdot T \cdot m_{\mathrm{cat}}} \cdot 0.5 \tag{4}$$

The determination of the proportion of nitrogen was done by equation (5). From the measured conversion of NO by the QMS setup X_{NO} and the formation of N_2O by the IR setup Y_{N_2O}, it was possible to calculate the nitrogen formation Y_{N_2}, Intervall [0,1].

$$Y_{N_2} = X_{NO} - Y_{N_2O}, [0,1] \tag{5}$$

This procedure was chosen because it turned out to be more precise than the correction of the MS signal 28 m/z with the CO content monitored by the IR setup and the fragmentation pattern of CO_2 and N_2O. The calculation of the selectivity toward nitrogen was done by equation (6).

$$S_{N_2} = \frac{Y_{N_2}}{X_{NO}} \cdot 100 \quad (6)$$

Table S3. Temperatures at a mass specific consumption rate of $z = 1.5 \cdot 10^{-6} \frac{mol}{s \cdot g}$ for CO and NO in each catalytic cycle for the LCM37 catalysts.

Sample	Cycle	T_z CO / °C	T_z NO / °C
LCM 37	1	335	270
	2	290	240
LCM 37	1	305	275
0.18 wt% Pd	2	285	255
LCM 37	1	235	205
0.86 wt% Pd	2	235	200
Pd/alumina	1=2	230	215

Table S4. Temperatures at a mass specific consumption rate of $z = 1.5 \cdot 10^{-6} \frac{mol}{s \cdot g}$ for CO and NO in each catalytic cycle for the LCM55 catalysts.

Sample	Cycle	T_z CO / °C	T_z NO / °C
LCM 55	1	280	265
	2	235	215
LCM 55	1	250	240
0.18 wt% Pd	2	225	205
LCM 55	1	235	225
0.86 wt% Pd	2	240	220
Pd/alumina	1=2	230	215

APPENDIX C: XRD characterization of the LCM37 samples

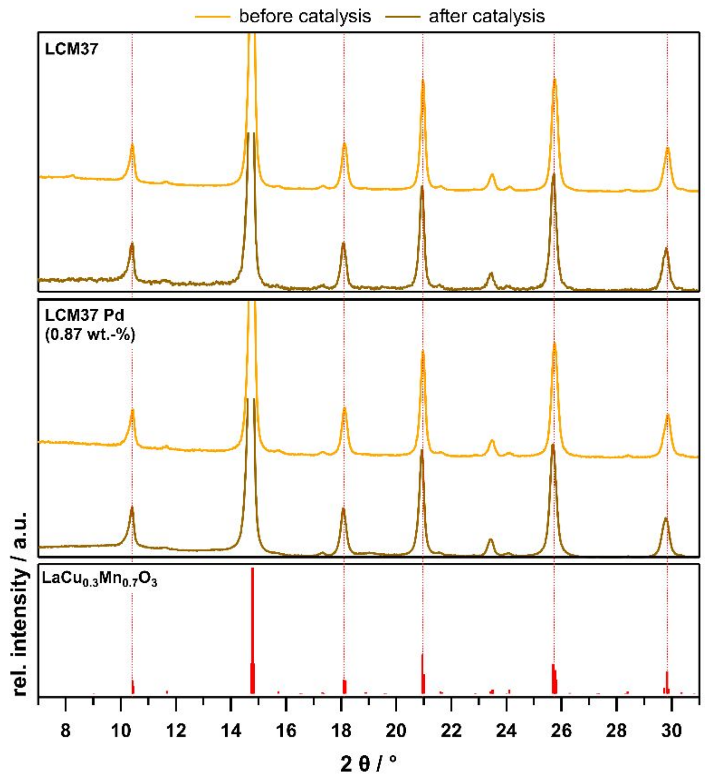

Figure S3. XRPD of LCM37(top), Pd-doped LCM37(middle) and the reference (bottom). Before (orange) and after catalysis (brown) respectively.

APPENDIX D: Determination of Cu(0)+Cu(I) vs. Cu(II) from XPS data with a peak fitting model according to Biesinger et al.

Here an example of the procedure is presented. Only Cu(II) species show major shake-up features. The main peak area (A1 and A2) representing Cu(0)+Cu(I) and Cu(II) and the shake-up feature area (B1 and B2) were used to calculate the amount of Cu(0)+Cu(I) (see Eq. (7)). The factor (A2/B) in Eq. 7 is determined by the pure LCM37 b.c. (before catalysis) sample and the Pd-doped LCM37 b.c. sample assuming the presence of Cu(II) only and amounts to 1.28 and 1.09 respectively.

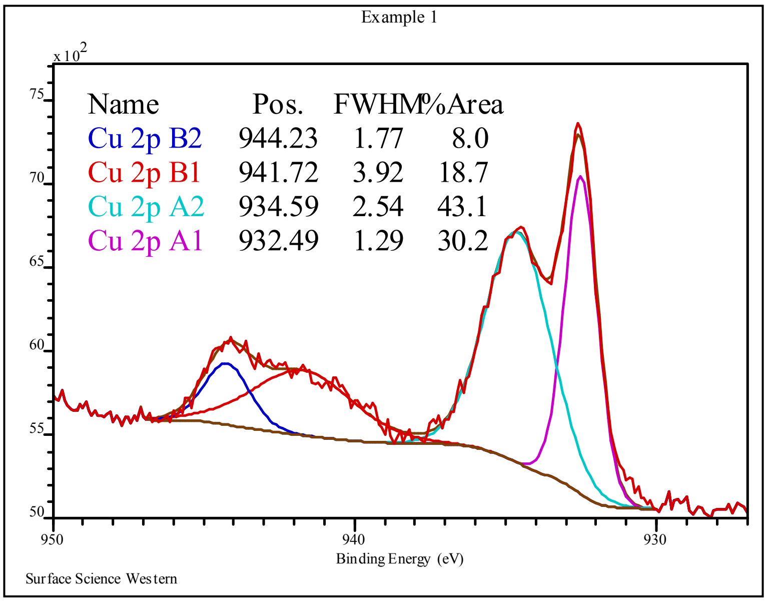

Figure S4. Example of a peak fitting model for the determination of Cu(0)+Cu(I)[4].

$$\% \, Cu(0) + Cu(I) = \frac{\text{A1}}{A+B} \cdot 100 = \frac{\text{A} - \text{A2}}{A+B} \cdot 100 = \frac{\text{A} - (\text{A2}/\text{B}) \cdot \text{B}}{A+B} \cdot 100 \qquad (7)$$

The peak fitting was performed using the CASA XPS Software (Version 2.3.19 PR1.0). The number of peaks, the positions, the FHWM relations and area relations were adopted from the following publication[4,5]. All constraints and relations are summarized in Table S5. The background was corrected using the Shirly function.

Table S5. Constrains and relations for the peak fitting in CASA XPS according to Biesinger et al.

Peak name	Position (eV)	Std.Dev. (+/- eV)	FWHM max (eV)[c,d]	Area relations (%)
2p 3/2 main peak (A)				
Cu (0) [b]	932,61	0,21		
Cu (II)[a]	933,80	0,50	3,1	64
shake-up peak (B)				
Cu (II) 1[a]	941,09	0,90	1,8	13
Cu (II) 2[a]	942,69	0,70	3,5	24

[a] GL(30) peak profiles for all oxide peaks. [b] GL(90) peak profile. [c] FWHM range for all metallic Cu peaks: 0.9 to 1.3 eV. [d] values for 20 eV Pass Energy.

Results of the XPS measurements of pure and Pd-doped LCM37 catalysts, from the respective fitting procedure evaluating the amount of Cu(0)+Cu(I) vs. Cu(II):

Table S6. Calculated values for the amounts of Cu(0)+Cu(I) and of Cu(II) (in at%) on the catalyst surface before to after catalysis using the peak fitting model according to Biesinger et al.

Sample	Cu(0)+Cu(I) /at%	Cu(II) /at%
LCM37 b.c.	0	100
LCM37 a.c.	45	55
LCM37 b.c. 0.86 wt% Pd	0	100
LCM37 a.c. 0.86 wt% Pd	58	42

APPENDIX E: Structure and morphology characterization of LCM37 with and without Pd by TEM

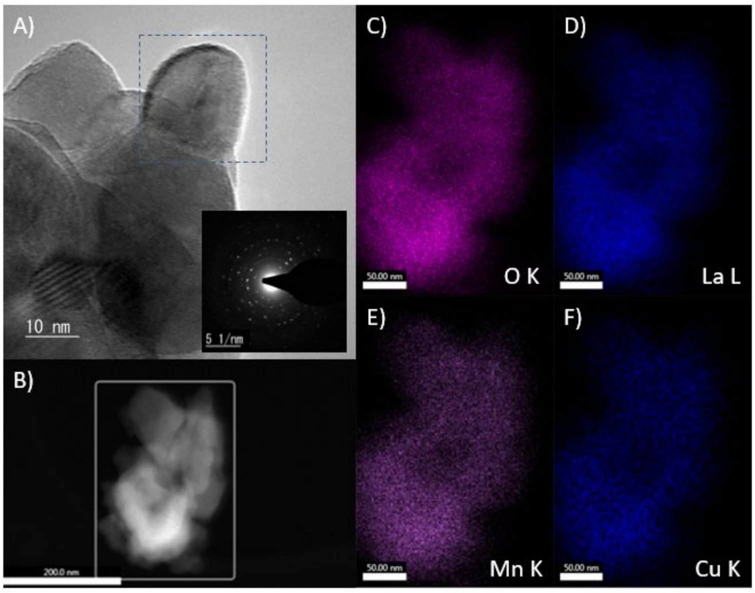

Figure S5. (A) HRTEM BF image of the LCM37 catalyst after synthesis and the according diffraction pattern as inset. (B) HAADF image of the EDX mapped particles , recorded in STEM mode. (C) EDX map of the oxygen K-line and the lanthanum L-line in (D). Panel (E): manganese and (F): copper, both K-lines.

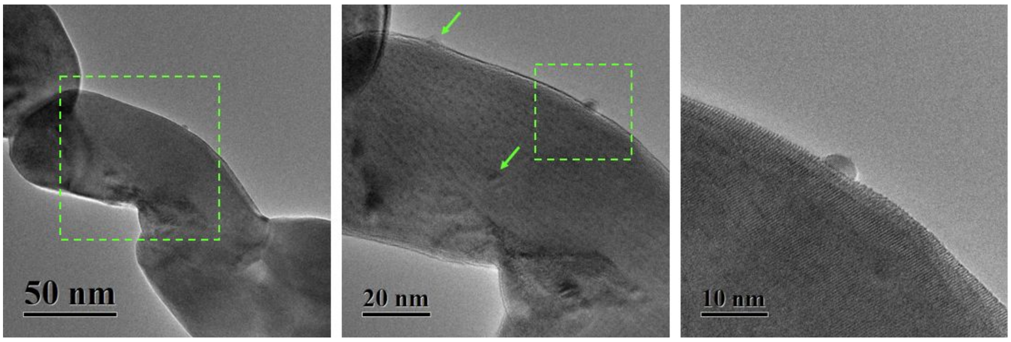

Figure S6. HRTEM images recorded under different magnifications of the LCM37 catalyst after NO+CO reaction, showing most likely exsolved Cu(0) particles (green arrows and box)

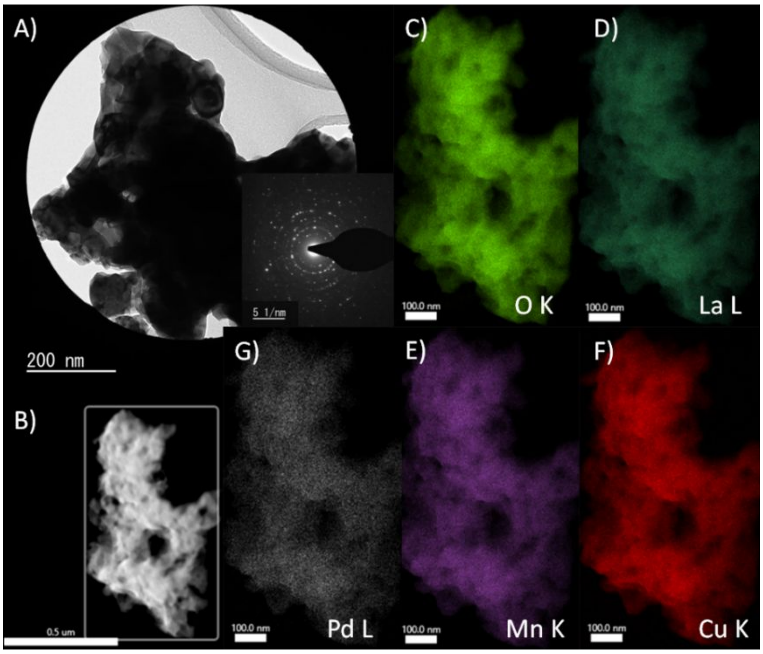

Figure S7. (A) TEM bright-field image of the Pd-doped LCM37 ($La(Cu_{0.3}Mn_{0.7})_{0.98}Pd_{0.02}O_{3-\delta}$) catalyst after synthesis, with the SAED region displayed in the circular section and the according diffraction pattern as inset. (B) HAADF image of the EDX mapped particles, recorded in STEM mode. (C) EDX map of the oxygen K-line and the lanthanum L-line in (D). Panel (E): manganese and (F): copper both K-lines. In panel (G), the EDX map of the palladium L-line is shown.

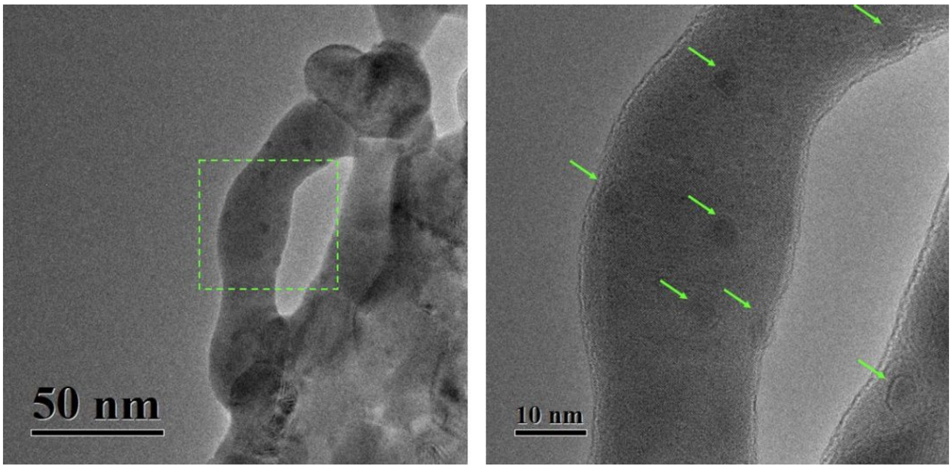

Figure S8. HRTEM images of the Pd-doped LCM37 catalyst recorded under different magnifications after NO+CO reaction. The green box illustrates the zoom region in the right panel. The arrows point at exsolved Cu-nanoparticles from the perovskite lattice.

APPENDIX F: Structure and morphology characterization of LCM55 with and without Pd by TEM

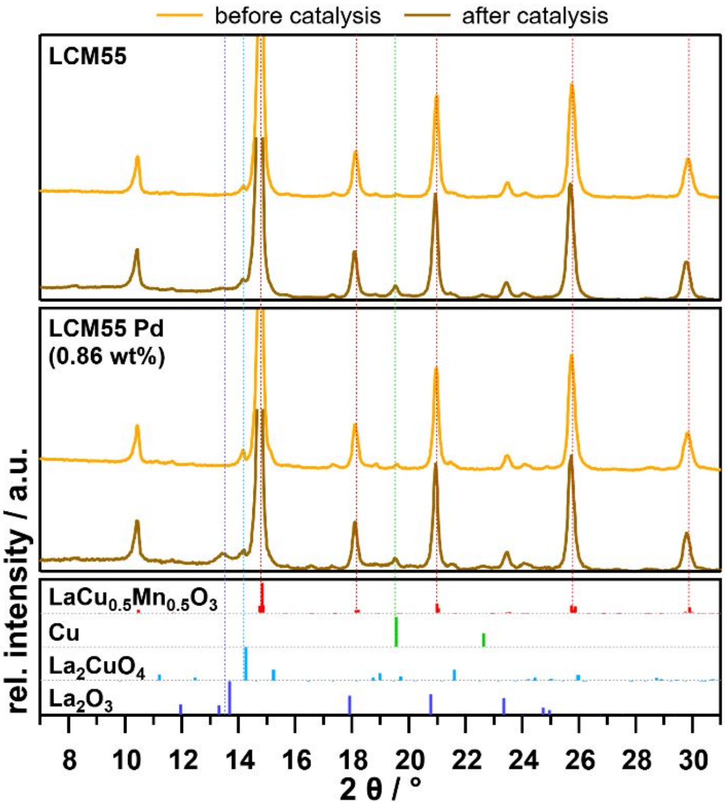

Figure S9. PXRD patterns recorded on LCM55 and Pd-doped LCM55. Orange and brown patterns indicate the measurements before and after catalysis, respectively. Reference patterns are highlighted in the lower section and were taken from ICDD and ICSD.

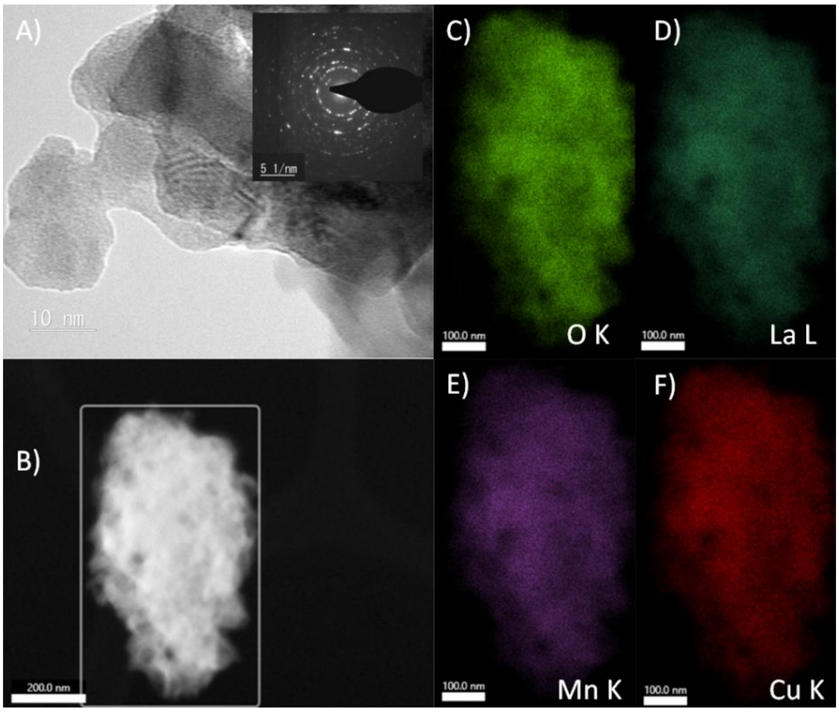

Figure S10. (A) TEM bright-field image of the LCM55 catalyst after synthesis and the corresponding diffraction pattern of the entire particle as inset. (B) HAADF image of the EDX mapped particles recorded in STEM mode. (C) EDX map of the oxygen K-line and the lanthanum L-line in (D). Panel (E): manganese and (F): copper, both K-lines.

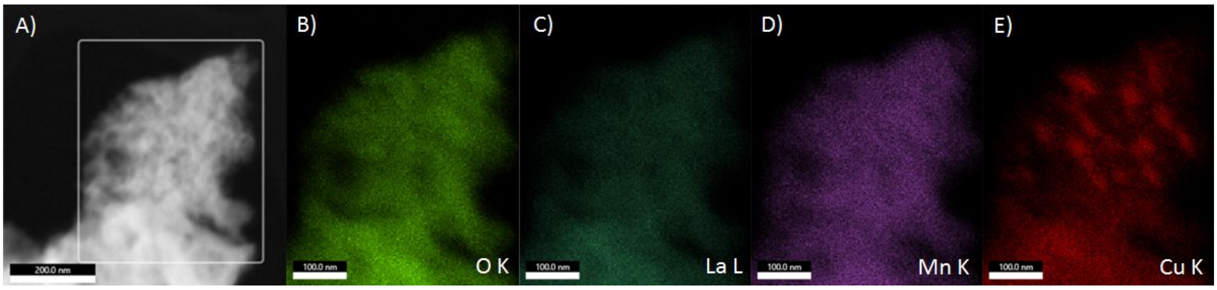

Figure S11. (A) HAADF image of the EDX mapped particle of undoped LCM55. (B) EDX map of the oxygen K-line and the lanthanum L-line in (C) as well as the manganese K-line in (D). In panel (E,) the copper K-line map yields Cu-clusters.

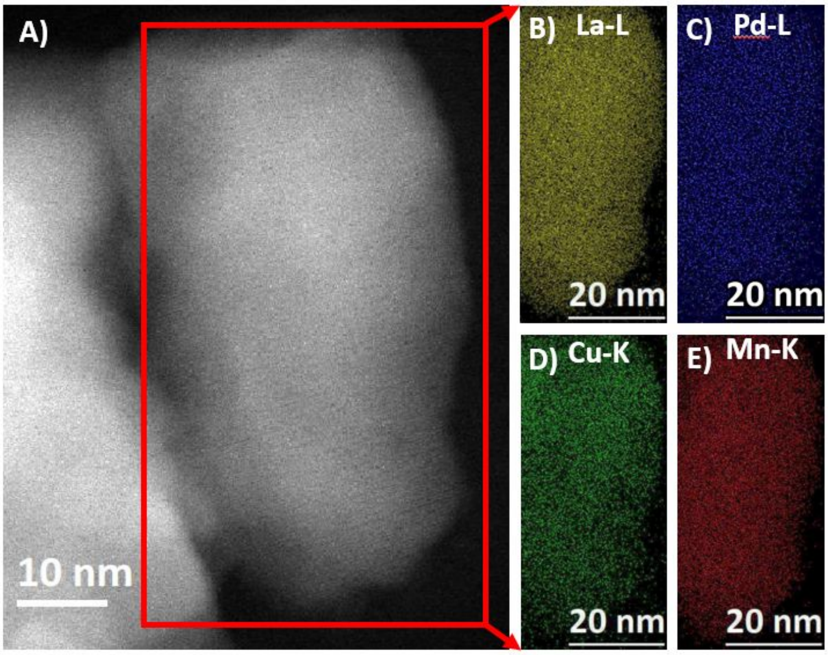

Figure S12. (A) ADF image of the Pd-doped (0.86 wt%) LCM55 catalyst after synthesis, where the rectangle region has been used for EDX analysis. Panel (B): La and (C): Pd both L-lines. Panel (D): Cu and (E): Mn, both K-lines.

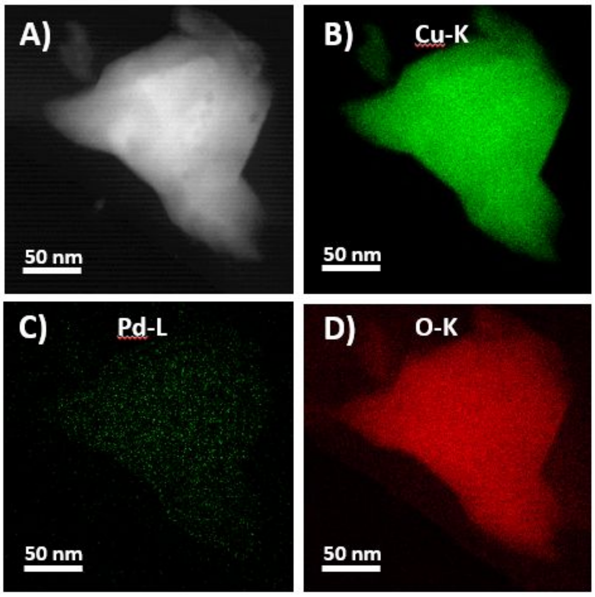

Figure S13. Cu/Pd nano-particles found in Pd-doped LCM55 catalyst after synthesis, with (A) ADF image and (B to D) EDX maps of the Cu K-line the Pd L-line and the O K-lines, respectively.

APPENDIX G: XPS measurements of pure and Pd-doped LCM55 catalysts, with the respective fitting procedure to evaluate the amount of Cu(0)+Cu(I) vs. Cu(II)

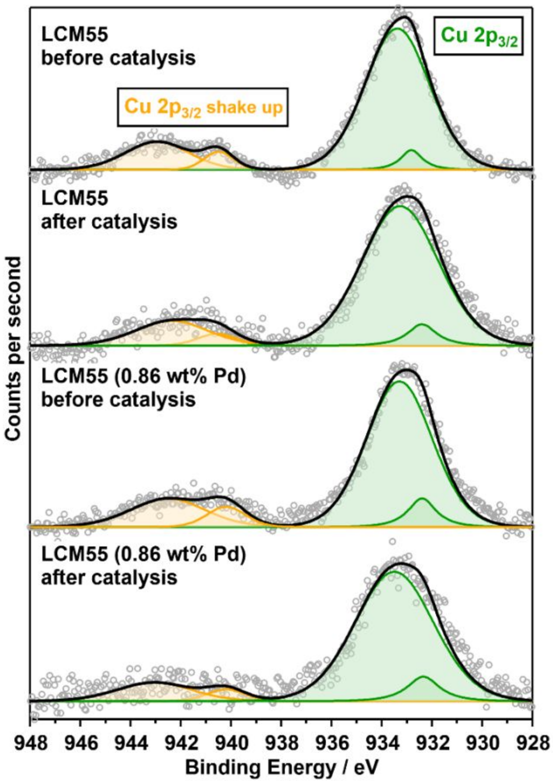

Figure S14. The XPS measurements of the Cu $2p_{3/2}$ region (gray circles) were evaluated with the peak fitting model described in Appendix C (orange curves: Cu(II) in the shake-up feature; green curves: Cu(II) and Cu(0)/Cu(I) in the main peak; fit envelope: black) to investigate the oxidation state of copper. From top to bottom: LCM55 before and after catalysis and Pd-doped LCM55 before and after catalysis.

Table S7. Calculated values for the amounts of Cu(0)+Cu(I) and of Cu(II) on the catalyst surface before to after catalysis using the peak fitting model according to Biesinger et al.

Sample	Cu(0)+Cu(I) /at%	Cu(II) /at%
LCM55 b.c.	55	45
LCM55 a.c.	62	38
LCM55 0.86 wt% Pd b.c.	55	45
LCM55 0.86 wt% Pd a.c.	70	30

APPENDIX H: BET specific surface area measurements of undoped and Pd-doped LCM-based catalysts

Table S8. BET specific surface area measurements of LCM-based catalysts, before catalysis.

BET	LCM37	LCM37 0.86 wt% Pd	LCM55	LCM55 0.86 wt% Pd
specific surface area/ m^2g^{-1}	16	6	11	5

APPENDIX I: XPS investigation of the Cu/SiO_2 reference catalyst

The pre-catalyst exhibits a clear Cu(II) state (Figure S15, top panel). Pre-reduction (350 °C, 3 h, 5% H_2 in He) causes a pure metallic Cu(0) state (Figure S15, middle panel). After NO+CO reaction we detect a more oxidized and therefore broadened Cu $2p_{3/2}$ peak compared to the pre-reduced catalyst and additionally a slightly emerging shake up feature, indicating a Cu(II) species (Figure S15, bottom panel). The detected counts at around 929 eV in the pre-reduced Cu/SiO_2 catalyst can be related to an interaction of Cu with SiO_2 under reducing conditions. Such an interplay can lead to a shift of the Cu 2p binding energy compared to bulk Cu, which has been reported for copper on zeolite materials[6].

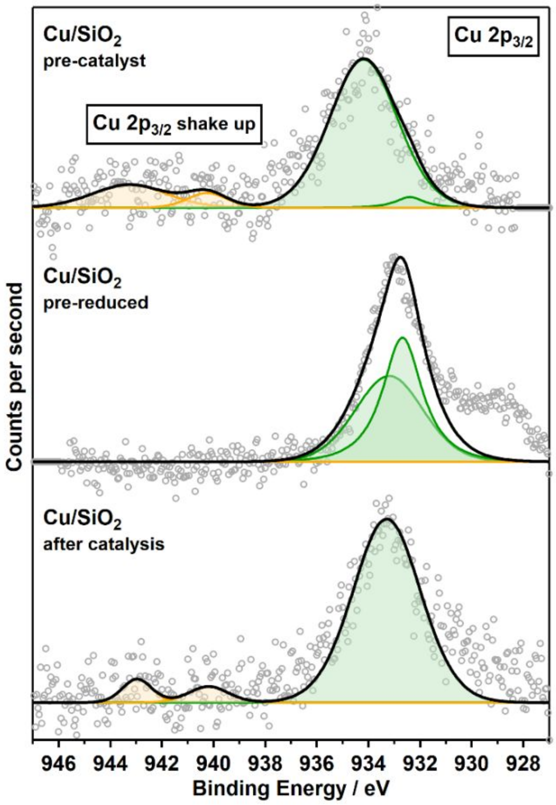

Figure S15. The XPS measurements of the Cu $2p_{3/2}$ region (gray circles) were evaluated with the peak fitting model described in Appendix C (orange curves: Cu(II) in the shake-up feature; green curves: Cu(II) and Cu(0)/Cu(I) in the main peak; fit envelope: black) to investigate the oxidation state of copper. From top to bottom: Cu on silica pre-catalyst, pre-reduced and after catalysis.

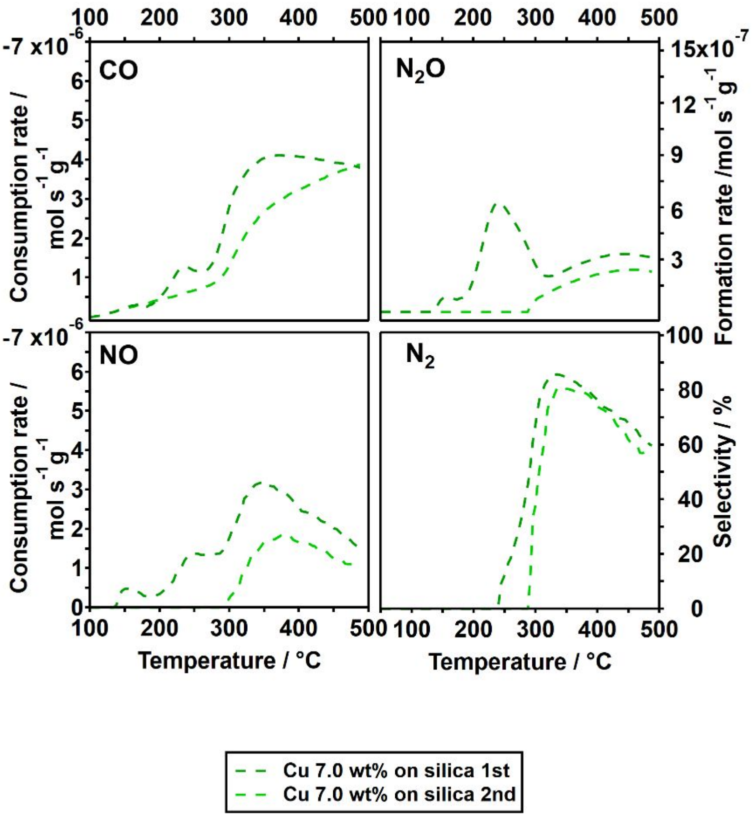

Figure S16. Consumption-rate profiles of CO and NO, the intermediate formation-rate profiles of N2O and the respective N2-selectivity profiles illustrate the catalytic deactivation of Cu-silica (dashed line, dark green 1st and light green 2nd cycle).

APPENDIX J: Reflex shift data evaluation for undoped and Pd-doped LCM-based catalysts

According to a Gaussian fit, Figure S17 and Table S9 show the shift of the main reflex at a 2θ position of approximately 14.7°.

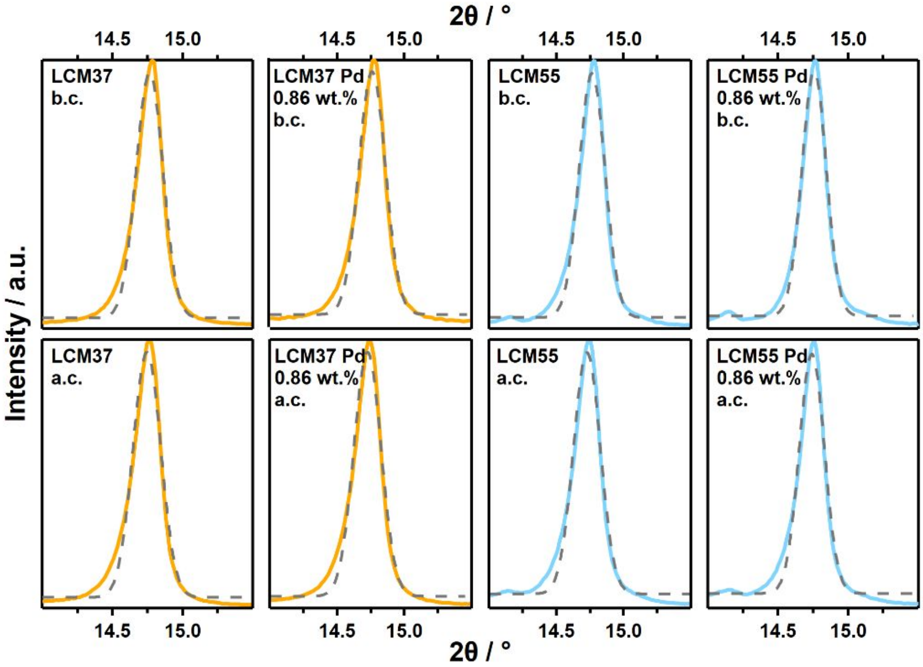

Figure S17: Fit (gray dashed line) of the main reflex at a 2θ position of approximately 14.7° for the undoped and Pd-doped LCM37 (orange) as well as LCM55 (blue) catalysts before (top row) and after (bottom row) the NO+CO reaction.

Table S9. Fit parameters of the main reflex at 2θ of ≈ 14.7° including the reflex positions before and after catalysis, the respective reflex shift and the full width at half maximum before and after catalysis.

	LCM37	LCM37 Pd 0.86 wt.%	LCM55	LCM55 Pd 0.86 wt.%
position b.c. / °	14.763	14.759	14.764	14.756
position a.c. / °	14.744	14.725	14.727	14.740
reflex shift / °	0.019	0.034	0.037	0.016
width b.c. / °	0.136	0.141	0.137	0.123

width a.c. / °	0.141	0.144	0.142	0.127

According to a Gaussian fit, Figure S18 and Table S10 show the shift of a side reflex at a 2θ position of approximately 29.8°.

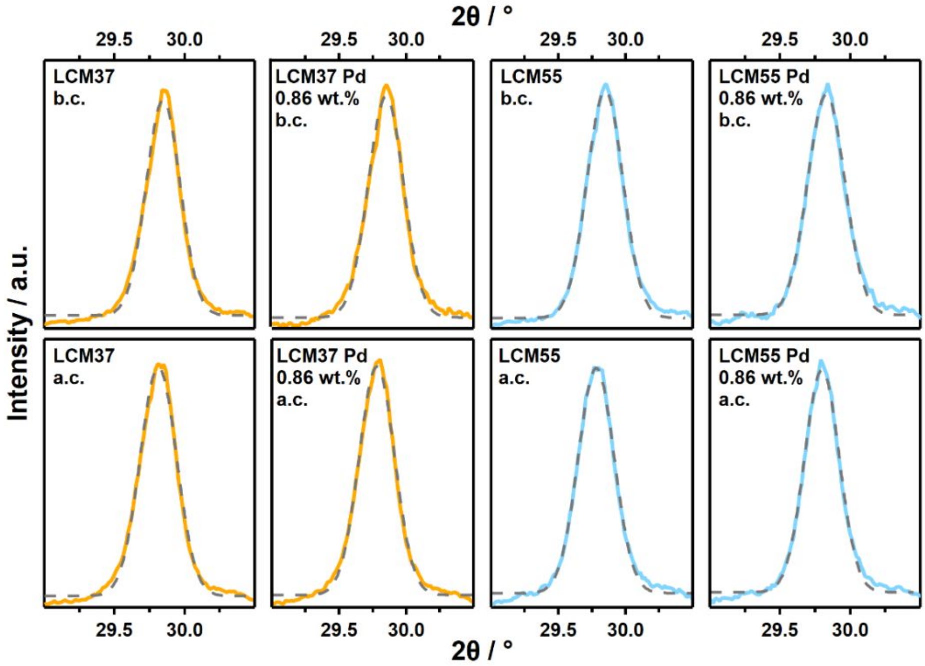

Figure S18. Fit (grey dashed line) of a side reflex at a 2θ position of approximately 29.8° for the undoped and Pd-doped LCM37 (orange) as well as LCM55 (blue) catalysts, before (top row) and after (bottom row) the NO+CO reaction.

Table S10. Fit parameters of a side reflex at a higher 2θ range (≈ 29.8°), including the reflex positions before and after catalysis, the respective reflex shift and the full width at half maximum before and after catalysis.

	LCM37	LCM37 Pd 0.86 wt.%	LCM55	LCM55 Pd 0.86 wt.%
position b.c. / °	29.846	29.847	29.846	29.828
position a.c. / °	29.812	29.780	29.781	29.798
reflex shift / °	0.034	0.067	0.065	0.030
width b.c. / °	0.173	0.186	0.190	0.188

width a.c. / °	0.184	0.187		0.189	0.175

APPENDIX K: Catalytic characterization of La_2CuO_4

From the derived data we conclude a negligible activity compared to the LCM-based catalysts (Figure S19).

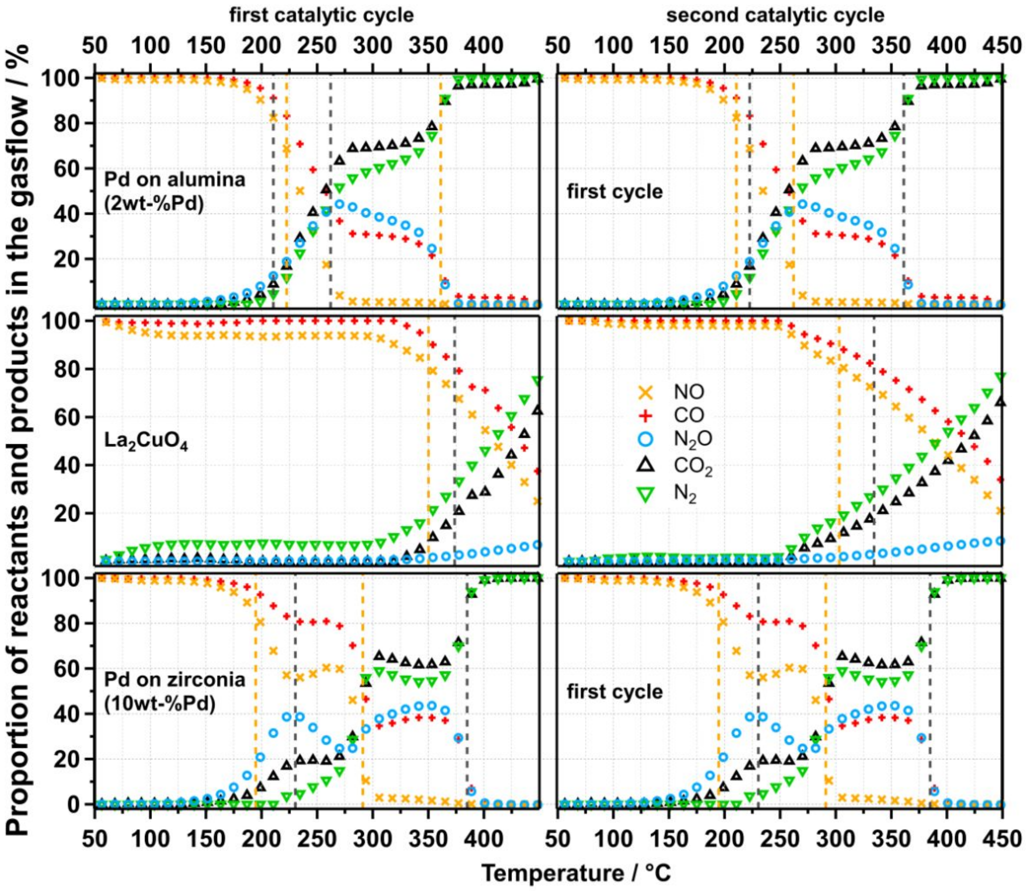

Figure S19. Catalytic measurement of La_2CuO_4 in the middle panel, top and bottom panels show activity/selectivity data of Pd on alumina and Pd on zirconia as reference catalysts, respectively. The vertical dashed lines show the temperatures for 15 % and 85 % conversion in orange for NO and in black for CO_2.

APPENDIX L: Long-term measuement of LCM55.

To evaluate the effect of Cu nanoparticle aggregation on the catalytic performance we heated the most active Pd-free catalyst LCM55 to the lowest temperature where full conversion were ensured. A loss of catalytically active sites is supposed to be a result of sintering and is followed by a lack of performance. As N_2 and CO are interfering in the mass spectrometer, CO_2 as a main product gas was utilized to monitor the activity over time (96 h = 4 days). Figure S20 illustrates the deactivation of LCM55 in an isothermal (350 °C) long-term measurement, starting with 100 % formation of CO_2 the signal decreases over four days to 82 %.

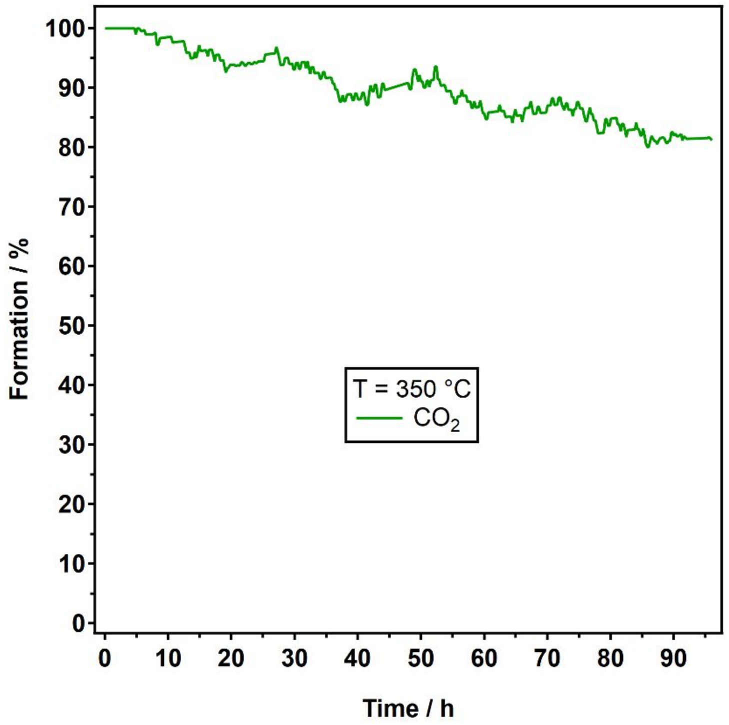

Figure S20. Long-term measurement of LCM55 at 350 °C isothermal conditions in NO + CO atmosphere (1% each in He). The green trace corresponds to the formation of CO_2 over measuring for 96 h.

The sintering effect is relatively moderate and activity can be expected to converge to a constant but still reasonable level after long-term operation. A 15% loss of active interfacial sites would correspond to a growth of the average particle diameter form 10.0 nm to 10.6 nm, according to the particle density model outlined in section A.

APPENDIX M: *In situ* XP spectra of the LCM-based Cu LMM auger region, Cu reference substance investigations and data evaluation of the *in situ* XP spectra of the LCM-based Cu $2p_{3/2}$ region

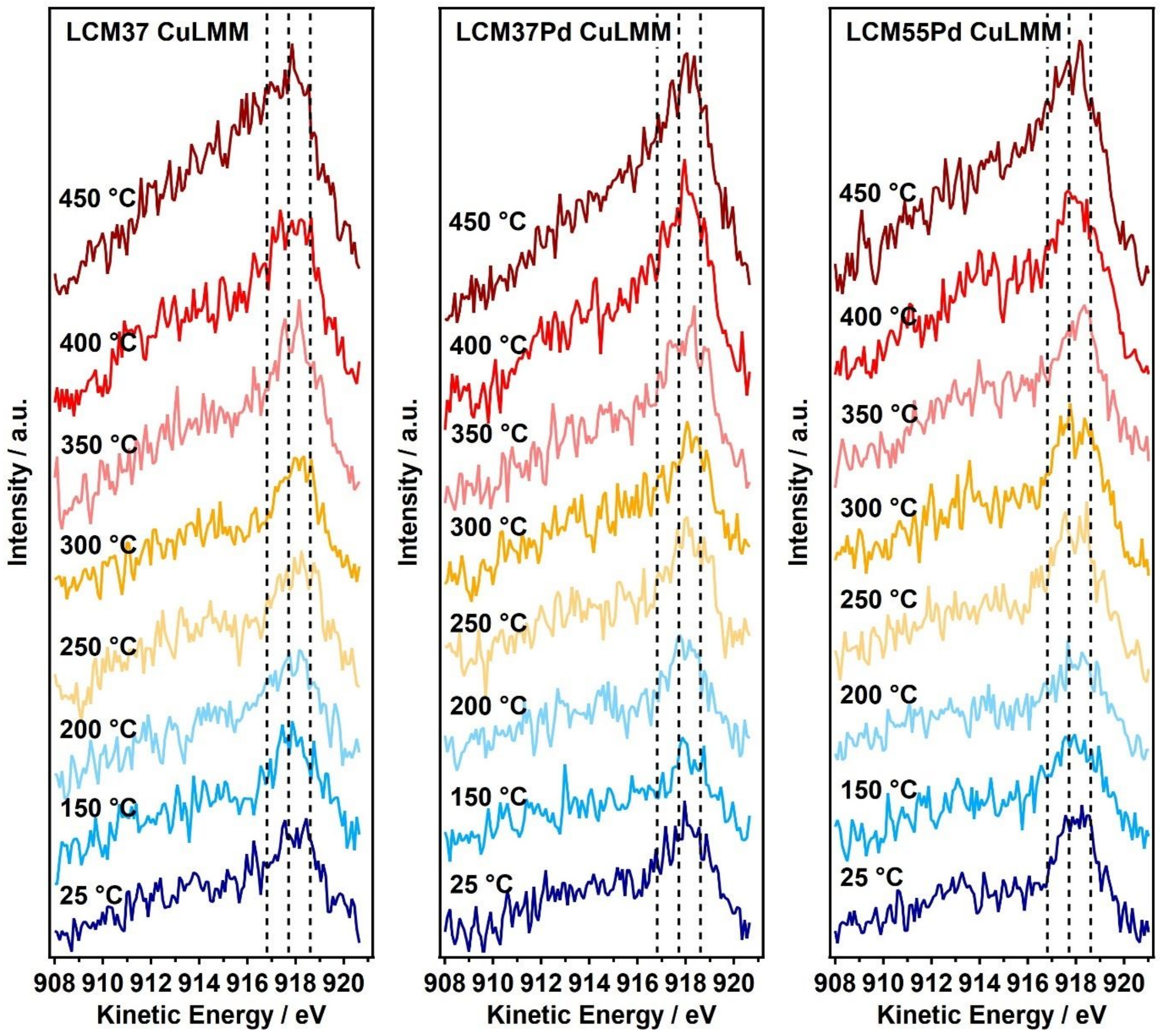

Figure S21. *In situ* XP spectra for pure LCM37 (left panel), 0.86 wt% Pd-doped LCM37 (mid panel) and 0.86 wt% Pd-doped LCM55 (right panel) catalysts of the Cu LMM auger region recorded at 8 isothermal temperature steps between 25 °C (dark blue) and 450 °C (dark red) in NO+CO atmosphere (0.3 mbar each). The vertical dashed lines indicates the position in kinetic energy (KE) of the peak maximum of the respective Cu reference substances (Cu(II): KE = 917.7 eV; Cu(I): KE = 916.8 eV; Cu(0): KE = 918.6 eV).

XP spectra and results derived from the Cu reference substances (metallic Cu, Cu_2O and CuO) – Cu $2p_{3/2}$ and Auger region

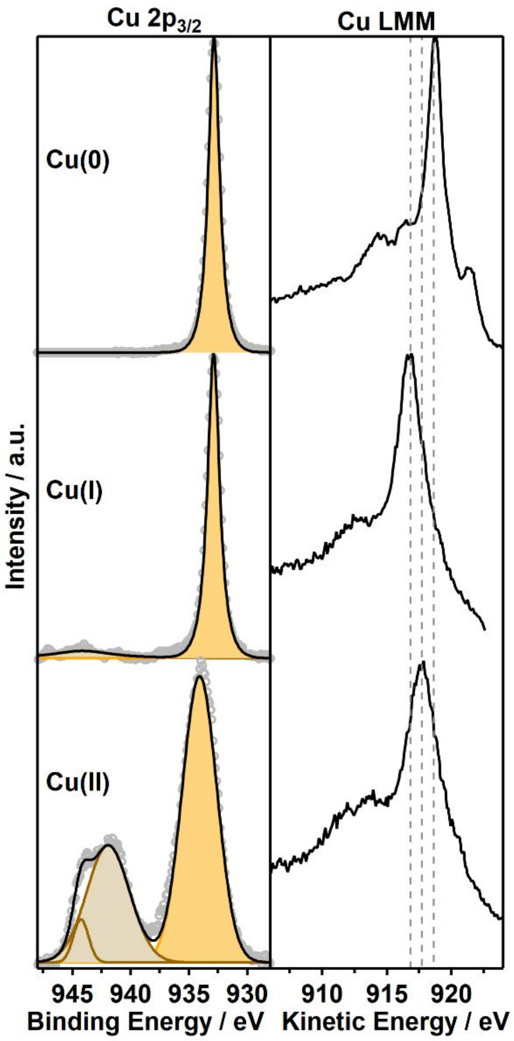

Figure S22. XP spectra of Cu-reference substances recorded with the same instrument as used for the *in situ* NAP XPS investigations of LCM-based catalysts. Cu, Cu_2O and CuO prepared and transferred by avoiding air contact in glovebox and glovebag respectively, were measured under UHV conditions (10^{-9} mbar regime). Metallic Cu was additionally Ar-sputter cleaned ($5 \cdot 10^{-5}$ mbar Ar, Filament current 3 A, Emission current 6 mA, Energy 2 keV) in a preparation chamber, directly before transfer and recording into the analysis chamber under UHV.

Table S11. Evaluated peak position and full-width-half-maximum (FWHM) for each Cu-reference substance and applied deviation used as constrains for the peak fitting in CASA XPS of all recoded *in situ* spectra and investigated samples.

Reference substance	Reference Position BE[a] Cu $2p_{3/2}$ (eV)[b]	Constrained Deviation (+/- eV)	Reference FWHM (eV)[c]	Constrained Deviation (+/- eV)	Reference Position KE[d] Cu LMM (eV)
Cu (0)	932.9	0.1	1.1	0.7 to 1.2	918.6
Cu (I)	932.9	0.1	1.2	0.7 to 1.2	916.8
Cu(II)	934.0	0.5	3.0	2.3 to 2.9	917.7

[a] Binding Energy [b] GL(30) peak profiles for Cu(II)-oxide peaks. GL(90) peak profile for Cu(I) and Cu(0) species [c] for 50 eV Pass Energy [d] Kinetic Energy

XPS fitting and normalization of the Cu $2p_{3/2}$ intensity to La $3d_{5/2}$

To compare catalysis to the peak area of the Cu(0)+Cu(I) and Cu(II) contributions of the four catalysts the respective Cu $2p_{3/2}$ peak areas were normalized using the La $3d_{5/2}$ intensities, as the contest of La is fixed in each sample.

Table S12. Normalization of the peak area of the Cu $2p_{3/2}$ region to the La $3d_{5/2}$ region in more detail the ratio of Cu(0)+Cu(I) to La $3d_{5/2}$ and Cu(II) to La $3d_{5/2}$ derived from the measurments in each temperature step.

	**LCM37**		**LCM37 Pd 0.9 wt%**		**LCM55**		**LCM55 Pd 0.9 wt%**	
**T / °C**	**Cu(0)+(I) / La $3d_{5/2}$**	**Cu(II) / La $3d_{5/2}$**	**Cu(0)+(I) / La $3d_{5/2}$**	**Cu(II) / La $3d_{5/2}$**	**Cu(0)+(I) / La $3d_{5/2}$**	**Cu(II) / La $3d_{5/2}$**	**Cu(0)+(I) / La $3d_{5/2}$**	**Cu(II) / La $3d_{5/2}$**
25	0,2	7,7	0,0	7,1	1,3	20,2	2,2	19,7
100	0,2	8,0	0,3	8,8	1,5	21,6	2,9	15,4
150	0,2	7,6	0,2	7,8	1,4	26,7	3,7	17,1
200	0,5	8,3	0,4	8,5	3,1	25,3	3,7	17,3
250	0,6	8,2	1,6	7,9	2,8	22,0	4,9	17,2
300	0,6	7,9	1,3	6,2	3,2	20,6	5,4	16,4
350	2,5	7,7	1,8	8,7	3,5	18,1	4,7	18,2
400	2,8	9,4	5,5	10,1	4,4	18,7	6,6	18,2
450	4,1	10,6	7,8	8,8	8,6	17,8	12,1	16,5
475					17,3	17,0		
500					17,2	18,7		

APPENDIX N: Further NAP-XPS measurement to monitor the Cu state in the second catalytic cycle.

To pinpoint the amount of metallic Cu evolves in a second catalytic cycle, we cooled down the catalyst in the applied NO+CO mixture (0.3 mbar each) to room temperature and carried out a second stepwise (50 °C) heat up from 100 °C to 500 °C. This procedure was accomplished for the most active Pd-free catalyst LCM55. In Figure S23 the recorded Cu $2p_{3/2}$ XP-spectra for each temperature step are waterfall plotted, whereby the left panel shows the first and the middle panel the second cycle respectively. In the right panel the ratio of Cu(0/I) to La evaluated for each T-step is presented and shows a very similar Cu(0/I)/La ratio after cooling down from 500 °C (cooling rate 10 °C/min).

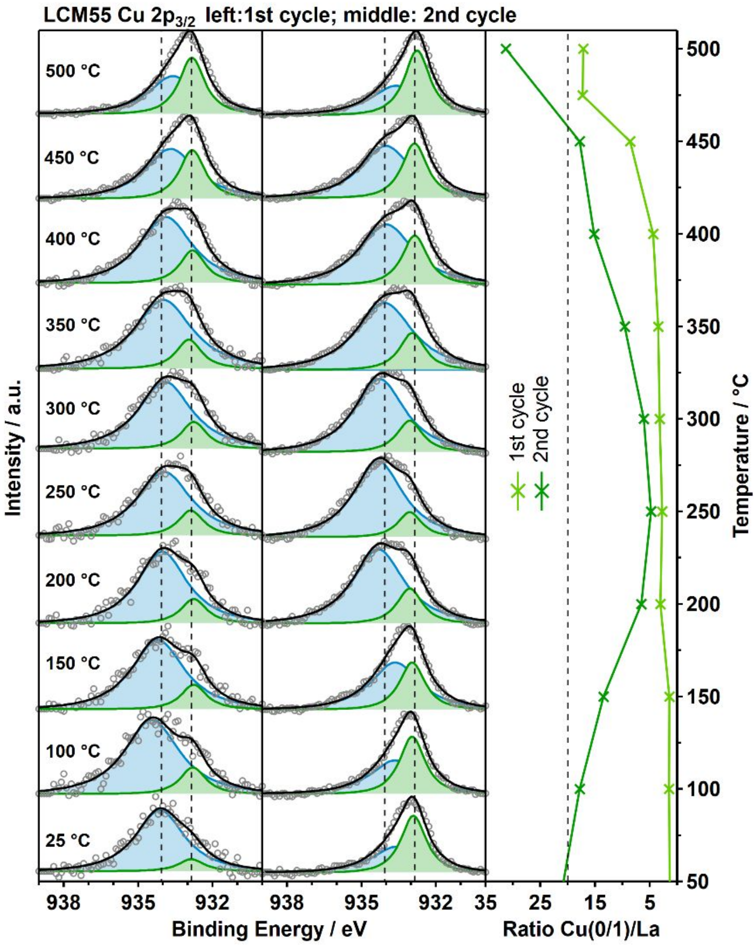

Figure S23. Cu $2p_{3/2}$ XP spectra recorded in situ under CO + NO atmosphere (0.3 mbar each) for 10 isothermal temperature steps between 25 °C and 500 °C for pure LCM55. The left panel shows the first and middle panel the second catalytic cycle. Experimental data: gray circles; colored fit components: blue

shaded Cu(II) and green shaded Cu(0/I); vertical dashed lines: BE reference values for Cu(II) 934.06 eV and Cu(0/I) 932.86 eV see Supporting Information, Appendix K; fit envelope: black line. In the right panel the La $3d_{5/2}$-normalized integral intensity of Cu(0) (light and dark green line with crosses, determined from the green shaded peak fittings) is plotted against the temperature for both cycles respectively.

Upon the second heat-up the oxidation state of Cu shifts from Cu(0/I) to Cu(II). In the catalytic active temperature region between 200 °C and 300 °C we detect an area of a stable Cu(II)/Cu(0/I) ratio manifesting itself as the active state of Cu during catalysis. Conclusively, this result strongly supports our proposed reaction mechanism showing an intermediate partial oxidation of metallic Cu during the conversion of NO and CO. Non the less by further increasing the temperature to 500 °C a significant higher amount of Cu(0/I) was observed. We suggest that higher temperatures enhance both the Cu mobility resulting in a more pronounced Cu-particle growth and the reaction kinetics suppressing the detection of the intermediate oxidation of metallic Cu sites.

REFERENCES

1. C.S. Fadley "Basic Concepts of X-ray Photoelectron Spectroscopy" from "Electron Spectroscopy, Theory, Techniques, and Applications" C.R. Brundle and A.D. Baker, Eds., Pergamon Press, **1978**, Volume 11, Chapter 1

2. Powell, C.J. and A. Jablonski, NIST Electron Elastic-Scattering Cross-Section Database SRD 64. Vol. Version 4.0. **2002**, Gaithersburg: National Institiue of Standards and Technology.

3. Powell, C.J. and A. Jablonski, NIST Electron Effective-Attenuation-Length Database SRD 82. Vol. Version 1.3. **2011**, Gaithersburg: National Institiue of Standards and Technology.

4. Biesinger, M. C., Advanced analysis of copper X-ray photoelectron spectra. *Surf. Interface Anal.* **2017**, 49 (13), 1325-1334.

5. Biesinger, M. C.; Lau, L. W. M.; Gerson, A. R.; Smart, R. S. C., Resolving surface chemical states in XPS analysis of first row transition metals, oxides and hydroxides: Sc, Ti, V, Cu and Zn. *Appl. Surf. Sci.* **2010**, 257 (3), 887-898.

6. Janas, J.; Gurgul, J.; Socha, R. P.; Dzwigaj, S., Effect of Cu content on the catalytic activity of CuSiBEA zeolite in the SCR of NO by ethanol: Nature of the copper species. *Appl. Catal. B* **2009**, 91 (1-2), 217-224.

AUTHOR CONTRIBUTIONS

Christoph W. Thurner: (lead) Sample preparation, catalytic tests, XRD and NAP-XPS experiments, data analyzation and evaluation, writing of original draft

Nicolas Bonmassar: (supporting) TEM investigations and analysis

Daniel Winkler: (supporting) XPS investigations and support concerning the data analyzation, contribution in NAP-XPS investigations and data evaluation

Lander Haug: (supporting) Contribution in NAP-XPS investigations

Kevin Ploner: (supporting) Support in sample preparation, XRD measurements and data evaluation

Parastoo Delir Kheyrollahi Nezhad: (supporting) Catalytic test of side phase La_2CuO_4

Xaver Drexler: (supporting) Long-term measurement of LCM55

Asghar Mohammadi: (supporting) Catalytic test of side phase La_2CuO_4

Peter van Aken: (supporting) Administration concerning the TEM investigations, validation

Julia Kunze-Liebhäuser: (supporting) Administration concerning the XPS investigations

Aligholi Niaei: (supporting) Administration concerning the sample preparation

Johannes Bernardi: (supporting) TEM investigation after sample synthesis

Bernhard Klötzer: (supporting) Project administration, support in writing of original draft, validation

Simon Penner: (supporting, corresponding) Project administration, support in writing of original draft, validation
